# Advances in Molecular Regulation of Prostate Cancer Cells by Top Natural Products of Malaysia

**DOI:** 10.3390/cimb45020099

**Published:** 2023-02-09

**Authors:** Jose M. Prieto, Mohd Mukrish Mohd Hanafi

**Affiliations:** 1 School of Pharmacy, University College London, London WC1N 1AX, UK; 2 School of Pharmacy and Biomolecular Sciences, Liverpool John Moores University, Liverpool L3 3AF, UK; 3 Institute of Bioproduct Development, Universiti Teknologi Malaysia, Skudai 81310, Malaysia

**Keywords:** prostate cancer, natural products, cytotoxicity, apoptosis, cell migration, cell invasion

## Abstract

Prostate cancer (PCa) remains both a global health burden and a scientific challenge. We present a review of the molecular targets driving current drug discovery to fight this disease. Moreover, the preventable nature of most PCa cases represents an opportunity for phytochemicals as chemopreventive when adequately integrated into nutritional interventions. With a renovated interest in natural remedies as a commodity and their essential role in cancer drug discovery, Malaysia is looking towards capitalizing on its mega biodiversity, which includes the oldest rainforest in the world and an estimated 1200 medicinal plants. We here explore whether the list of top Malay plants prioritized by the Malaysian government may fulfill the potential of becoming newer, sustainable sources of prostate cancer chemotherapy. These include *Andrographis paniculate, Centella asiatica, Clinacanthus nutans, Eurycoma longifolia, Ficus deltoidea, Hibiscus sabdariffa, Marantodes pumilum (*syn*. Labisia pumila), Morinda citrifolia, Orthosiphon aristatus,* and *Phyllanthus niruri*. Our review highlights the importance of resistance factors such as Smac/DIABLO in cancer progression, the role of the CXCL12/CXCR4 axis in cancer metastasis, and the regulation of PCa cells by some promising terpenes (andrographolide, Asiatic acid, rosmarinic acid), flavonoids (isovitexin, gossypin, sinensetin), and alkylresorcinols (labisiaquinones) among others.

## 1. Introduction 

Cancer incidence and mortality rapidly grow despite significant drug discovery and clinical practice advances. Lung cancer is the most frequent cancer and the leading cause of cancer death among males, followed by prostate and colorectal cancer (for incidence) and liver and stomach cancer (for mortality) [1]. In the last decade, incidence remained high, with an estimated 1.1 and 1.4 million men worldwide diagnosed with prostate cancer in 2012 and 2021, respectively. However, the magnitude of increasing incidence and decreasing prostate cancer mortality has been attenuated [2]. The high number of cases in more developed nations could be due to the already common practice of prostate-specific antigen (PSA) screening and subsequent biopsy [3]. In the United Kingdom, prostate cancer contributed to 46,690 (13%) of new cases and 11,287 deaths annually. In addition, 54% of prostate cancer cases in the UK are diagnosed in males aged 70 and over every year [4]. According to the statistics provided by the National Cancer Registry [5] of Malaysia, prostate cancer ranked ninth overall and is the fourth most frequent cancer (7.3% of all cancers) diagnosed in men. Although the incidence of prostate cancer is more prevalent in Western countries, the number of prostate cancer cases has also grown in different parts of the world. Genome-wide association studies have identified around 100 genetic loci associated with prostate cancer risk in Western populations but only a dozen in Japanese and the Chinese populations [6], thus reflecting a specific baseline protection for male Asians. 

It is therefore hypothesized that diet and lifestyle largely contribute to prostatic carcinogenesis [7], and younger generations’ rapid adoption of an urban Western lifestyle may be the reason for the rapid rise of prostate cancer in Asian countries such as Japan and Hong Kong [8,9]. Recently, chronic epithelial cell injury accompanied by innate and adaptive inflammatory responses have been highlighted as the underlying initial molecular pathophysiological steps in prostate cancer [10]. 

On the one hand, inherited susceptibility and sex steroid hormones causing epithelial damage leads to cancer as men age. On the other hand, there are dietary components, chiefly heterocyclic amines, able to exert cell and genome damage resulting in prostate inflammation [11]. Historically, androgens were the central paradigm for pathogenesis and therapeutics in prostate cancer. However, epidemiological studies show that breast and prostate cancers generally coincide worldwide [12]. Experimental studies support the hypothesis that estrogens might cause both diseases [13], and researchers brand 17β-Oestradiol as a “genotoxic hormone” able to both induce DNA damage and keep genome integrity [14].

Whether Asian traditional dietary customs are either protecting or contributing to prostate cancer may be a question of a delicate balance between the ubiquitous presence of plant-derived estrogens (phytoestrogens) [15] and certain popular Asian culinary ingredients able to directly counteract the deleterious effects of heterocyclic amines [16]. To further complicate matters, the same phytoestrogens may act as both cytotoxic/antitumoral/chemopreventative agents [17,18,19,20], as well as favor cancer cell proliferation [21]. The intestinal microbiota and its differential ability to convert some isoflavones into even more potent estrogen-like compounds may be a new lead for solving the conundrum of contradictory epidemiological studies on soy consumption, prostate, and breast cancers [22].

## 2. Aims and Methods

Our aim here is to review the potential of phytochemicals as either preventative therapy or chemotherapy for prostate cancer. To this end, we will present an introduction of the main known molecular and genetic factors of prostate cancer; a discussion of some mechanisms underlying cancer progression that may afford new targets; an overview of the possible commercial and clinical routes for natural products to become part of prostate cancer prevention and/or therapy; and a literature review of the anti-prostate cancer potential of the top ten Malaysian natural products. Finally, our conclusions will try and inform future research and development.

The sources are mainly PubMed (https://pubmed.ncbi.nlm.nih.gov/) and Google Scholar (https://scholar.google.com/) and were accessed between 2017 and 2022. There were not strict inclusion/exclusion criteria. In Section 1, Section 2, Section 3, Section 4, Section 5, Section 6 and Section 7 (incl.), the authors preferred more established theories and mechanisms of clinical relevance related to chemotherapy for prostate cancer. We gave less attention to immunotherapies or new molecular developments not yet translated into therapeutic approaches. Section 8 reviews Malaysian plants focusing on their effects on prostate cancer cells. We report on data obtained with different cancer cells only if relevant for their potential use in prostate cancer research. The molecular mechanisms reported from these studies also informed the author’s choice of topics in Section 3 and Section 6.

## 3. Molecular and Genetic Factors of Prostate Cancer

### 3.1. Chromosomal Abnormalities and Oncogenes

Chromosomal abnormalities associated with prostate cancer include the deletion of 8p, 10q, 13q, and 16q, as well as the gains of 7p, 7q, 8q, and Xq chromosomes. These allelic abnormalities have been reported by Nupponen and Visakorpi [23], Hughes and co-workers [24], and Shi and co-workers [25]. Further allelic loss has been reported by Rubin and Rubin [26], Shen and Abate-Shen [27], and Fraser and co-workers [28], which include the loss of 6q, 7q, 17p, and 18q. 

The *MYC* is a well-known oncogene that plays an essential role in regulating cellular proliferation, differentiation, and apoptosis. This oncogene is located at 8q24 and another amplified region of 8q [29,30,31,32]. The overexpression and amplification of this oncogene have been detected in prostate cancer cells, especially in the metastatic stage [32,33,34]. Besides *MYC*, the *RAS* family of oncogenes are the most common oncogenes in human cancer. However, in prostate tumors, mutations in the ras genes (*HRAS*, *KRAS,* and *NRAS*) are relatively uncommon [35,36], except in the rare ductal form of the disease. Morote and co-workers [37] have reported that overexpression of the *ERBB2* gene is a frequent event in prostate cancer. The *ERBB2* gene, or *Her-2/neu,* is from a family of genes that provide instructions for producing growth factor receptors. These growth factors are essential in stimulating cell growth and division. *ERBB2* gene amplification will result in the overproduction of ErbB2 protein, which can cause cells to grow and divide continuously, leading to uncontrolled cell division, one of the hallmarks of cancerous tumor progression [38,39]. Another oncogene that plays a significant role in the progression of prostate cancer is *Bcl-2*. Several authors have reported the overexpression of *Bcl-2*, especially in recurrent tumors [40,41,42,43,44]. However, this event did not seem to happen due to the amplification of the genes [23,43]. Bcl-2 inhibits apoptosis of the prostate cancer cells subjected to androgen deprivation, allowing the cancerous cells to survive without the required hormone. 

### 3.2. Androgen Receptor

The androgen receptor (AR) is a type of nuclear receptor that is encoded by a single copy gene located on the X-chromosome (Xq11.2-q12), which consists of 919 amino acids in length, but this can vary depending on the poly-glutamine, poly-glycine, and poly-proline repeats of variable lengths [45]. AR signaling is crucial as it plays a critical role in prostate function and differentiation in the growth and progression of prostate cancer [45,46]. AR activity is regulated by two major ligands, testosterone, and dihydrotestosterone (DHT). DHT, which has a ten times higher binding affinity to the AR, is the primary androgen bound to the AR. The binding of DHT to the AR promotes the recruitment of protein kinases, which leads to the phosphorylation of several serine residues. This process is essential as it serves many functions, such as protection from proteolytic degradation, stabilization, and transcriptional activation [46]. The transactivation of the AR is vital as it regulates specific gene targets involved in cell growth and survival [47]. The rate of cell proliferation and the rate of cell apoptosis are balanced in normal prostate epithelium. However, this balance is lost in prostate cancer, leading to the formation of tumor cells [48]. Since prostate cancer growth is highly dependent on androgen, androgen-ablation therapy has always been the most effective treatment for prostate cancer at an early stage. However, this therapy only manages to delay tumor progression by 18–24 months, followed by the development of a lethal drug-resistant stage known as castration-resistant prostate cancer (CRPC). Visakorpi and co-workers [31] have reported that in CRPC patients, the frequent amplification of chromosome Xq in recurrent tumors has led to the overexpression of the AR after androgen deprivation therapy. This type of chromosomal amplification is rarely seen in primary tumors. The overexpression of the AR overcomes the decreased levels of circulating androgens in hormone-independent prostate cancer, thus allowing the cancer cells to continue growing even in a deficient level of androgen left in serum after castration [31,49]. The overexpression of AR also produces a receptor that is more sensitive to a low androgen level, or that can be activated by other types of steroids, such as adrenal androgens, estrogens, progestins, and anti-androgens used in the management of the disease [50]. 

### 3.3. Metastasis

Prostate cancer is one of the most malignant types of cancer in men. It can spread to distant sites, including bones, lymph nodes, lungs, liver, and brain. However, prostate cancer frequently metastasizes to the bone marrow [51]. Almost 90% of advanced prostate cancer patients suffer from pathologic fractures, spinal cord compression, and pain due in part to deregulated cycles of osteoblastic and osteolytic resorption/formation driven by the growing tumor mass [52,53]. Although the mechanisms that account for the tendency of prostate cancer cells to metastasize to the bone have not yet been elucidated, they may include direct vascular pathway, highly permeable sinusoids, chemotactic factors produced by bone marrow stromal cells, and the synthesis of growth factors necessary to support cell survival and the proliferation of ‘seeded’ cancer cells [54,55]. Taichman and co-workers hypothesize that metastatic prostate carcinomas may use the hematopoietic model to localize to the bone barrow. In this model, chemokines, a group of molecules known to play significant roles as activators and chemoattractants, including CXC chemokines such as CXCL12 and its receptor CXCR4, appear to be critical molecular determinants for the events in this model [56,57]. They also showed that the CXCL12/CXCR4 chemokine axis was activated in prostate cancer metastasis to the bone [58]. They also confirmed that CXCR4 expression is related to increasing tumor grade and showed that CXCL12 signaling through CXCR4 triggers the adhesion of prostate cancer cells to bone marrow endothelial cells [59]. Similar studies by other researchers also suggest that the CXCL12/CXCR4 axis plays a similar role in other tumors metastasizing to the marrow. For example, Mueller and co-workers [60] reported that the CXCL12/CXCR4 axis plays a central role in regulating metastasis by showing that normal breast tissues express little CXCR4 receptors compared to breast neoplasms, which express high levels of CXCR4. Furthermore, this study also shows that using an antibody that could block the CXCR4 receptor could prevent the spread of tumor cells to the lungs and lymph nodes.

Apart from that, the metastatic progression of prostate cancer is also closely associated with two genes, namely E-cadherin (*CDH1*) and *KAI1* genes. The expressions of both genes are significantly reduced in metastatic prostate cancer cells [61,62]. However, this was not caused by allelic loss but rather by post-transcriptional events regulated by *p53*. Therefore, a loss of *p53* function in the late stages of tumor progression could cause the downregulation of these two genes with subsequent metastasis [63].

### 3.4. Arachidonic Acid Metabolism

Besides the abnormalities in chromosomes, the presence of oncogenes, and the overexpression of specific receptors, cancer cells commonly overexpress key enzymes of the arachidonic acid metabolism (mainly COX-2 and 5-LOX). COX-2 is overexpressed in practically every premalignant and malignant condition involving the colon, liver, pancreas, breast, lung, bladder, skin, stomach, head, neck, and esophagus [64,65,66,67,68,69,70,71]. Interestingly, human prostate cancer cells are known to generate 5-lipoxygenase (5-LOX) instead. 5-LOX is a type of enzyme in humans encoded by the *ALOX-5* gene. Ghosh and Myers [72] reported that chemical constituents such as arachidonic acid, omega-6, and polyunsaturated fatty acid stimulate prostate cancer cell growth via the 5-LOX pathway. This has been corroborated by Yang and co-workers [73], who also point towards 12-LOX. The expression of 5-LOX is usually restricted to specified immune cells such as neutrophils, eosinophils, basophils, and macrophages.

In contrast, most non-immune body cells do not express 5-LOX unless at the onset of certain diseases such as asthma, arthritis, psoriasis, and cancer [74,75,76,77]. The 5-LOX plays a significant role in chemotaxis in these cells [74]. Ghosh and Myers [72] reported that the inhibition of 5-LOX would block the production of 5-LOX metabolites and trigger apoptosis in prostate cancer cells. The expression of 5-LOX in normal prostate glands is almost undetectable but is augmented in prostate tumor tissues. Therefore, this finding is significant for developing future therapeutic approaches for prostate cancer, as 5-LOX plays a critical role in the survival of prostate cancer cells. All this leads to the concept that 5-LOX may play a major role in the development and progression of prostate cancer and could be used as a promising target in prostate cancer therapy. Other abnormalities, including the amplification and overexpression of certain genes mentioned earlier, could also be used as a potential target in future prostate cancer therapy.

### 3.5. Angiogenesis

The tumor typically consists of cancer cells and stromal cells. These stromal cells face a hostile metabolic environment characterized by acidosis and hypoxia. Therefore, tumor development requires the supply of oxygen and nutrition, usually provided by the nearby blood vessels [78]. With this, the tumor growth is only limited to 1–2 mm in diameter (avascular phase) [79]. Therefore, the tumor needs an increased blood supply mainly provided by forming new blood vessels from pre-existing capillaries and venules to exceed the size limit. This process is called angiogenesis [79]. Like other tumor cells, prostate tumors overexpress the vascular endothelial growth factor (VEGF) [80]. The VEGF is a homodimeric glycoprotein that belongs to the first group of pro-angiogenic factors [81]. Other important pro-angiogenic factors include the fibroblast growth factor (FGF) [82], platelet-derived growth factor B (PDGF-B) [83], angiopoietins [82,83], growth-related oncogenes [84], tumor growth factor β (TGFβ) [85], and matrix metalloproteases (MMPs) [86].

The overexpression of the VEGF in prostate tumors will promote the development of tumor neovascularization, and this overexpression also correlates with increasing grade, vascularity, and tumorigenicity. Besides that, the receptor for the VEGF, VEGFRs, and α_5_β integrin were expressed by prostate cancer cells in vitro and prostate tumors in vivo, and their expression was elevated at sites of bone metastasis compared to the original prostate tumor. He and co-workers [87] reported that the angiogenic effect of the VEGF in the prostate tumor could be blocked by the inactivation of its receptor, the VEGFR. Without the VEGF or VEGFR, the prostate tumor cells would not be able to form sprouting capillaries. Therefore, this could be a potential target to stop the development and progression of prostate cancer.

Prostate-specific membrane antigen (PSMA) serum levels have been proposed to be a better prognostic value than PSA to evaluate the effectiveness of prostate cancer treatments, either via surgery, hormones, radiation, or chemotherapy [88]. Interestingly, PSMA overexpression is observed in the neovasculature of solid tumors but not in the vasculature of normal tissues [89]. The correlation between PSMA and VEGF expression in LNCaP-induced tumors reinforced its value as a marker for angiogenesis. Mechanistically, PSMA may modulate integrin [90] and the nuclear factor kappa B [91] signaling pathways. Some authors later attributed this correlation to the Mouse double minute 2 (*MDM2*), a negative regulator of the *p53* tumor suppressor, which regulates VEGF expression and angiogenesis, after gene profiling studies suggested a signalling interplay between *MDM2* and PSMA [92]. This view has been recently expanded after Watanabe and co-workers experimentally showed how PSMA-positive vesicles secreted from prostate cancer cells have the potency to transform vascular endothelial cells into an angiogenic state [93]. This has been followed by preliminary results on how they can induce PSMA-negative cells to secrete VEGF [94]. These new data open new views on how PSMA may induce pro-tumoral changes in the tumor microenvironment, thus supporting tumor progression.

## 4. Chemoprevention of Prostate Cancer

The idea of chemoprevention was initially introduced in the mid-1970s [95,96]. It consists of the administration of agents or substances—such as drugs or vitamins—to try and reduce the risk or delay the development or recurrence of cancer. Carcinogenesis in cancer development is a complex process that generally requires an extended period; therefore, chemoprevention could significantly inhibit or slow this process before cancer growth becomes clinically significant [97]. Chemoprevention is a type of preventive medicine divided into primary, secondary, and tertiary prevention. Primary prevention deals with the incidence of disease in an otherwise healthy individual. Secondary prevention aims to stop the progression of a disease to a clinically significant stage by focusing on the treatment given to an individual already with a premalignant condition. Finally, tertiary prevention focuses on preventing the recurrence or progression of disease in an individual previously treated with malignancy [98]. In the case of prostate cancer, chemoprevention is essentially aimed at primary and secondary prevention [98].

Many reasons support the use of chemoprevention as part of prostate therapy. As mentioned earlier in this section, carcinogenesis in cancer development occurs over time, which is also the case for prostate cancer. Carcinogenesis in prostate cancer is thought to consist of protracted multistep molecular processes affecting numerous pathways [99]. This molecular pathogenesis could lead to the development of pre-cancerous characteristics such as atypical small acinar proliferation (ASAP) and high-grade intraepithelial neoplasia (HGPIN) [100]. Both ASAP and HGPIN can be detected many years before the formation of the actual cancer mass in the prostate gland itself. These characteristics make prostate cancer an ideal target for primary chemoprevention. In addition, prostate cancer also has a prolonged latent disease state, therefore increasing the incidence of prostate cancer in elderly men aged between 60–79 years old [101]. This specific characteristic of prostate cancer makes it suitable as a target for secondary chemoprevention.

In chemoprevention, using low toxicity agents or substances is vital as it involves treating healthy individuals [102]. In prostate cancer, chemoprevention can prevent the transformation of normal cells or precursor lesions to cancerous cells (secondary prevention) and stop the growth of the existing tumor cells [103]. 5 Alpha-reductase inhibitors are one of the chemopreventives used in prostate cancer therapy. Inhibition of 5-α-reductase will decrease dihydrotestosterone (DHT) levels in prostate cancer tissues, thus lowering the androgenic stimulation to the prostate [95]. Several studies showed that the inhibition of 5-α-reductase in prostate cancer tumors grafted into an animal has significantly impeded tumor implantation and growth [104,105]. Finasteride is a type of 5-α-reductase inhibitor which lowers intraprostatic DHT levels while causing testosterone levels to increase slightly [106]. It was the first 5-α-reductase inhibitor to enter a human trial. Another 5-α-reductase inhibitor used for prostate cancer chemoprevention is dutasteride. Unlike finasteride, dutasteride is thought to have better efficacy in chemoprevention as it can inhibit both isoforms of 5-α-reductase [107]. A recent study by Andriole and co-workers [108] showed that dutasteride reduced the risk of prostate cancers and precursor lesions among men at increased risk of prostate cancer and benign prostatic hyperplasia (BPH) while improving many outcomes related to BPH. Other agents such as vitamin C, vitamin B1, vitamin B2, vitamin B3, calcium, zinc, protein, and selenium have been used to prevent prostate cancer growth. However, no clear association exists between these common dietary factors and prostate cancer [95]. In this study, characterization of the active extracts could lead to their use in prostate cancer chemoprevention in the future.

## 5. Clinical Management of Prostate Cancer

Treatments and prostate cancer management depend on several factors, such as the stage of the disease, age, general health, and a person’s view of potential treatments and their possible side effects. Due to the aggressiveness and the annoying side effects (erectile and urinary dysfunction) of most treatment options, discussions often focus on balancing therapy goals with the risk of lifestyle alterations. A combination of treatment options is highly recommended for managing the disease [109,110,111]. The management of prostate cancer can be divided into three categories: (1) management of localized prostate cancer, (2) management of locally advanced prostate cancer, and (3) management of metastatic prostate cancer.

Most patients diagnosed with localized prostate cancer will have an excellent prognosis after radical prostatectomy and/or radiation therapy. One-fifth of the patients opt out of surgery and will undergo ‘watchful waiting.’ However, 20–40% of the patients who went through primary therapy will experience biochemical relapse, with 30–70% of those developing metastatic disease within ten years after the introduction of local therapy [112,113,114,115]. The metastatic stage of prostate cancer is usually managed by the use of androgen-deprivation therapy, also known as hormone ablation therapy. A luteinizing hormone-releasing hormone (LHRH) agonist will improve the symptoms, but tumors invariably become hormone-independent (castration-resistant) and evolve to the progressive stage. Before the 1990s, patients with castration-resistant prostate cancer (CRPC) were generally treated with palliative approaches, as no other life-prolonging options were available. As prostate cancer is often associated with older men with limited bone marrow reserve and concurrent medical conditions, chemotherapy at this time was not recommended as it can further deteriorate the quality of life [116].

Tannock and co-workers [117] have reported that mitoxantrone (with prednisone) can improve quality of life and reduce bone pain besides lowering serum PSA levels in men with CRPC. This important discovery has led to using intercalating agent mitoxantrone as the initial standard of care for such patients. The cytotoxic Docetaxel replaced mitoxantrone as the first line of care in 2004 after the Southwest Oncology Group reported that its administration with estramustine significantly extended progression-free and overall survival for CRPC patients, when compared to mitoxantrone with prednisone [118]. However, until 2010, physicians have no other life-prolonging second-line options after the failure of docetaxel. On June 17 of the same year, the US Food and Drug Administration (FDA) approved the use of cabazitaxel for CRPC patients previously treated with a docetaxel-containing regime (US Food and Drug Administration), thus changing from the stabilization of microtubule to the inhibition of the assembly as the underlying mechanism of therapeutic action. Until recently, other therapies, including Enzalutamide (Xtandi^®^), Abiraterone (Zytiga^®^), and Radium-233 (Xofigo^®^) have been used for the management of CRPC patients. Enzalutamide and Abiraterone are a new type of hormone therapy for men with prostate cancer that no longer responds to hormone therapy or chemotherapy. Enzalutamide works by blocking the hormone testosterone from reaching the prostate cancer cells whereas Abiraterone works by stopping the production of testosterone in the body. Without the availability of this hormone, prostate cancer cells will cease to grow wherever they are in the body.

The introduction of a new concept of systemic disease: high-risk or high-volume vs. low-risk and low-volume disease [119] opened a new clinical scenario for sequence therapies using the above-discussed drugs [120,121]. All major clinical associations have reflected these breakthroughs in treatment in updated professional guidelines. However, real-world adoption of such combination therapy protocols is still poor [122], compounded by mean nonadherence rates of 25% to 51% in patients prescribed oral prostate cancer therapies, with higher rates in older patients [123]. Mind–body therapies are also gaining momentum within the scientific community, such as yoga and Tai Ji Chuan/Qigong, all known to alleviate fatigue and improve the quality of life in PCa patients. Hypnosis and relaxation training showed to reduce nausea/vomiting and improve sleep and anxiety, thus increasing resilience to support oncological treatments [124]. Moreover, clinicians are revisiting the paradox of androgen-castration in the light of bipolar androgen therapy that involves rapid cycling from supraphysiological back to near-castration testosterone levels over a 4-week cycle [125].

## 6. Possible Therapeutic Targets for Prostate Cancer Based on the Major Hallmarks of Cancer

The six main hallmarks of cancer proposed initially by Hanahan and Weinberg [126] include sustaining proliferative signaling, evading growth suppressors, resisting cell deaths, activating invasion and metastasis, inducing angiogenesis, and enabling replicative immortality. The same authors later extended this list after considering the progress made in the past decade by adding two new hallmarks comprising the reprogramming of energy metabolism and the evasion of immune destruction [127]. These are the biological capabilities acquired by the tumor cells during their multistep development that enable them to become tumorigenic and ultimately malignant. Understanding these hallmarks is crucial to finding suitable therapeutic targets that can be used more effectively in cancer treatment. Blandin and co-workers [128] have reported that ß1 integrins, which are a type of matricellular receptor (extracellular matrix component), play an essential role in the modulation of these cancer hallmarks. Therefore, they may be one of the suitable therapeutic targets for cancer therapy. This study aims to find bioactive compound/s from plants that could modulate some cancer hallmarks, namely resisting cell death or evading apoptosis, activating invasion and metastasis, and inducing angiogenesis. These are the three main hallmarks of cancer that would be the focus of this study. The discovery of bioactive compound/s with the ability to modulate these three important hallmarks would be vital for the future of prostate cancer therapy.

### 6.1. Resisting Cell Death: Role of Smac/DIABLO in Cancer Progression

The ability of tumor cells to expand in numbers depends not only on the rate of cell proliferation but also on the rate of cell death, which generally occurs through apoptosis. This process is tightly regulated in normal cells to maintain cell population and immune system development [129]. However, disruptions and deregulations of the process lead to uncontrolled cell growth, a characteristic of tumor cells. Therefore, inducing cancer cells, apoptosis is essential in cancer therapy, such as prostate cancer therapy.

Two distinct pathways can activate apoptosis: the extrinsic pathway, mediated by death receptors (CD95 and TRAIL), or the intrinsic pathway, mediated by the mitochondria [130]. Stimulating death receptors such as CD95 or TNF-related apoptosis-inducing ligand (TRAIL) located on the plasma membrane activates the initiator caspase-8, which can cause a direct cleavage of downstream effector caspases such as caspase-3 [131]. Effector caspases such as caspase-3 will cause irreversible damage to the nuclear lamina, cleave the proteins that typically hold a DNA degrading enzyme (DNAse) in an inactive form, freeing the DNAse to cut the DNA in the cell nucleus and ultimately causing the dismantling of the cells quickly and neatly [132].

The mitochondrial-driven (intrinsic) pathway is initiated with the permeabilization of the outer mitochondrial membrane by pro-apoptotic members of the Bcl-2 family, such as Bax and Bak [133]. Proteins from the Bcl-2 family regulate this process (e.g., Bcl-2, Bcl-xL, Bcl-w, Bax, Bak, and Bok) together with mitochondrial lipids, and other proteins involved in metabolic flux as well as components of the permeability transition pore [133]. After the disruption of the outer mitochondrial membrane, the apoptogenic factors such as cytochrome *c*, apoptosis-inducing factor (AIF), TG (Smac/DIABLO), Omi/HtrA2, or endonuclease G are released into the cytosol from the mitochondrial intermembrane space [134,135]. For example, the release of cytochrome *c* activates caspase-3 and eventually leads to the dismantling of the cells neatly and quickly without causing any harm to the neighboring cells. 

Smac/DIABLO is a novel mitochondria-derived, pro-apoptotic protein important in sensitizing tumor cells to die by apoptosis [136,137]. It has a pro-apoptotic effect mediated by its interaction with the inhibitor of apoptosis proteins (IAPs) and the release of effector caspases from them. The IAP protein family that includes the XIAP, c-IAP1, and c-IAP2 blocks both intrinsic and extrinsic apoptotic pathways by binding to and inhibiting active caspases, thus stopping the caspase cascade [138]. The XIAP is the most potent among the IAPs due to the presence of three domains known as the baculoviral IAP repeat (BIR) domains. The BIR domains are fundamental in inhibiting the activity of active caspases [139]. Smac/DIABLO functions by neutralizing the caspase-inhibitory properties of the XIAP in the displacement of the XIAP from caspase nine by Smac/DIABLO, thus overcoming the ability of the XIAP to repress the activity of effector caspase, caspase-9, within the apoptosome complex [140]. Several studies have shown that Smac/DIABLO is crucial for mitochondrial-driven apoptosis in human multiple myeloma and prostate cancer cells [141]. The Smac peptide also enhances apoptosis-induced chemo or immunotherapeutic agents in the leukemic Jurkat cell line [142] and malignant glioma cells in vivo [143]. These findings prompted scientists to develop peptides derived from the NH2-terminal of Smac/DIABLO and small molecules that mimic the function of Smac/DIABLO as therapeutic agents to induce cancer cells death or increase the apoptotic effects of the chemotherapeutic agents [130]. Currently, small molecules such as AT-406, LCL-161, and Birinapant are all in the preclinical phase for their anticancer properties in combination with more specific chemotherapy drugs such as TRAIL and BRAF inhibitors [144]. 

### 6.2. Activation of Invasion and Metastasis: Role of CXCL12/CXCR4 Axis in Cancer Metastasis

Prostate cancer can spread to distant sites, including lymph nodes, lung, liver, and brain. However, the spread to the bone marrow is the most frequent and as a consequence, almost 90% of advanced prostate cancer patient suffer from pathologic fractures, spinal cord compression, and pain due in part to the deregulated cycles of osteoblastic and osteolytic resorption/formation driven by the growing tumor mass [52]. In addition, several reports have shown that the CXCL12/CXCR4 axis is involved in breast [60] and prostate [58,145] cancer cells’ metastasis to bone, where high expression of CXCR4, the receptor for the CXCL12 chemokine, is detected [146,147]. 

Chemokines are a superfamily of cytokine-like proteins that activate chemokine receptors by binding to them [148]. Over 50 chemokines have been identified and divided into four distinct families, namely CXC, CX3C, CC, and C. These divisions are made based on the positions of four conserved cysteine residues [149]. Previously, chemokines were associated with their inflammatory responses achieved through the stimulation of leukocyte chemotaxis during inflammation [150]; however, new pieces of evidence suggest that they are major regulators of cell trafficking and adhesion [151,152]. For example, CXCL12, or the stromal-derived factor 1 (SDF-1), is a CXC chemokine, and its receptor CXCR4 is reported to play a critical role in the spread of prostate cancer cells to the bone. 

The binding of CXCL12 to CXCR4 is said to initiate divergent signaling pathways that lead to multiple responses, including the phosphorylation of MEK/ERK signaling cascade and the activation of NF-κB, which is essential for tumor cell survival [148]. Moreover, Sun and co-workers [153] have reported that the levels of CXCL12 in human and mouse tissues were higher at the preferable sites of metastasis for prostate cancer cells, such as bone, liver, and kidney, when compared with tissues rarely affected by the tumor cells, including lung, tongue, and eye. In addition, higher expression of CXCL12 leads to the increased adhesion of prostate cancer cells in the endothelial cell monolayer and immobilized fibronectin, laminin, collagen [154], and osteosarcoma cells [58] due to the upregulation of α5 and β3 integrins. 

Apart from that, overexpression of the CXCR4 gene was documented in aggressive phenotypes of prostate tumor tissues, and prostate cancer patients with this phenotypic characteristic often have poor survival rates [155]. Sun and co-workers [153] have reported that in an in vivo study, the extent of bone metastasis in prostate cancer was limited by using a neutralizing antibody against CXCR4. These data clearly show that the CXCL12/CXCR4 axis plays a major role in prostate cancer cell progression. In our study, we investigate the potential of bioactive compound/s from plants to modulate this axis to reduce or prevent prostate cancer cell migration and invasion.

### 6.3. Induction of Angiogenesis: Role of VEGF in Angiogenesis

One of a malignant tumors’ most important phenotypic traits is their ability to have sustained angiogenesis. Angiogenesis is crucial as it ensures a continuous supply of oxygen, nutrients, growth factors, hormones, and proteolytic enzymes [156]. Without angiogenesis, the progression of malignant tumors to the distant site will not be possible. Although a report was made a century ago regarding the observation that increased vascularity can accompany tumor growth [157], it was not until 1939 that the first postulate about the existence of a tumor-derived blood vessel growth-stimulating factor that served to aid the formation of new blood supplies to the tumor was made by Ide and colleagues [158]. Six years later, Algire and co-workers [159] proposed that the rapid growth of tumor transplants depends on developing a rich vascular supply. This proposal was based on the observation that tumor growth started after an increase in the blood-vessel density using a transparent chamber technique on the mouse [159,160]. In 1971, Folkman proposed that finding an anti-angiogenesis factor could be an effective anticancer strategy [161], and this pioneering hypothesis has led to the understanding of several important angiogenic factors such as the epidermal growth factor (EGF), transforming growth factor (TGF)-α, TGF-ß, tumor-necrosis factor-α (TNF-α), and angiogenin [162]. However, their effects in experimental models could not always bet translated into physiological effects [163]. 

The vascular endothelial growth factor (VEGF), which is now known to be the main regulator of angiogenesis, was first described by Senger and co-workers [164] and then was successfully isolated and identified by Ferrara and colleagues in 1989 [165]. The VEGF is a homodimeric glycoprotein with an approximate molecular weight of 45 kDa. It belongs to a family of structurally related mitogens known as the platelet-derived growth factor (PDGF) [166]. The VEGF family consists of six secreted glycoproteins known as VEGF-A, VEGF-B, VEGF-C, VEGF-D, VEGF-E, and placenta growth factor (PlGF-1 and PlGF-2) all of which are derived from distinct genes [167]. 

VEGF-A plays a critical role in the regulation of angiogenesis [168], whereas the vascular effect of the other VEGFs in the family members remains fully characterized [169,170,171]. Initially, two VEGF receptors were identified on endothelial cells and characterized as the specific tyrosine kinase receptors VEGFR-1 and VEGFR-2 [172,173]. Several years later, another tyrosine kinase receptor known as VEGFR-3 was identified and has been found to play a significant role in lymphangiogenesis [174,175]. All VEGF family members have a different binding affinity towards these receptors, and this characteristic has helped to elucidate their specific functions. All VEGF-A isoforms bind to both VEGFR-1 and VEGFR-2, while the naturally occurring heterodimers of VEGF-A have a higher binding affinity towards VEGFR-2 [176,177]. 

Since VEGF-A can bind to both VEGFR-1 and VEGFR-2 receptors, questions have been asked about which receptor activation could lead to the direct effect on tumor cell angiogenesis. A consensus has been reached on this matter, as studies show that VEGFR-2 is the major mediator of the angiogenesis process [178]. Even though VEGFR-1 is said not to affect mitogenesis and angiogenesis directly, it might act as a decoy receptor that sequesters VEGF and prevents its interaction with VEGFR-2 [179]. 

Angiogenesis, also known as neovascularization, is very important in human cancers and is specifically linked to increased tumor growth and metastatic potential [180]. Overexpression of VEGF mRNA can be observed in various human tumors, including lung, breast, prostate, gastrointestinal tract, renal, and ovarian carcinomas, through in situ hybridization studies [181,182,183,184,185,186]. The expression of VEGF ligands in tumor cells supported the hypothesis that claimed that VEGF supports the growth of tumor cells not only by inducing angiogenesis, but also through their direct action on VEGFRs that creates a VEGF/VEGFR autocrine loop, which induces tumor growth arrest and apoptosis when disrupted [167]. Furthermore, studies conducted by Warren and co-workers [187] have demonstrated that in an in vivo mouse colon cancer model of liver metastasis, a significant decrease in the size of the tumor was achieved after the treatment with anti-VEGF monoclonal antibodies. Other studies also show that a significant decrease in tumor size and prolonged survival were achieved in mice bearing human leukemias xenograft after treatment with antibodies that function by blocking the VEGFR-2 receptor [188]. Therefore, the results from all these studies clearly demonstrate that angiogenesis is VEGF-dependent and finding bioactive compound/s that could block VEGFs and its receptors or both could reduce or prevent tumor cell angiogenesis.

## 7. Opportunities for Phytochemicals: Food or Drug?

The downside to recent advances in the therapy of PCa is that the treatment costs increase more rapidly than for any other cancer. Thus, the case for the preventative use of phytochemicals with experimental antitumoral activity may be considered a valid option for personalized protection in primary care as well as for advanced disease management at the level of secondary and tertiary care in the framework of predictive, preventive, and personalized medicine [189]. A successful example of this strategy is the saw palmetto berry, widely used for treating benign prostatic hyperplasia (BPH), often as an alternative to pharmaceutical agents. Although pathologically BPH is not considered a precursor for prostate cancer, approximately 83% of prostate cancer incidence occurs in men with BPH [190,191], and both conditions share common risk factors and respond to anti-androgen therapy. Saw palmetto is a palm plant commonly found along the Atlantic and Gulf coasts of the USA. The large berries produced by the plant are highly enriched in fatty acids, phytosterols, and flavonoids [192]. Numerous clinical trials had been conducted to evaluate the effectiveness of the extract of the fruits in treating BPH [193,194,195], and based on the positive results of these clinical trials, saw palmetto was developed as a whole extract with no single active ingredient, unlike any other conventional drug. It has been marketed as a dietary supplement to treat enlarged prostate. In continental Europe, saw palmetto is available as a full licensed medicine (Permixon^®^) but also available as a traditional medicine (THMP) (Prostasan^®^) as well as a food supplement without explicit indications but translating to the health-conscious consumer the hidden message of prostate health. Outside Europe, the market drastically splits into food or drugs, leaving saw palmetto manufacturers and consumers with the food supplement pathway as the only way to bring this efficacious natural product to the public. Approximately 2.5 million adults in the United States were reported to use saw palmetto in a National Survey conducted in 2002 [196]. It is also prevalent in Europe, as half of German urologists prefer prescribing plant-based extracts to synthetic drugs [197,198]. Saw palmetto is available in the market as an encapsulated capsule, powdered berries, and gel capsules containing the liquid extract. This extract is thought to treat BPH by inhibiting the 5-α-reductase enzyme [199,200], similar to finasteride, a popular chemopreventive used in prostate cancer therapy, as explained in Section 4. Several studies were conducted to investigate whether saw palmetto is active against prostate cancer. These studies showed that the lipidosterolic extract of saw palmetto appears to inhibit LNCaP, PC3, 267B-1, and BRFF-41T prostate cancer cell lines in vitro when treated with physiologically plausible doses [199,200,201]. The inhibition of the prostate cancer cell lines occurs through several different mechanisms, including apoptosis, necrosis, and growth inhibition [199,200]. However, no studies have been conducted to examine saw palmetto activity in animal models. The development of saw palmetto as an agent to promote prostate health could be an excellent example of how the active extracts of other plants can be introduced into the market shortly.

### 7.1. Research and Development of Anticancer Drugs from Natural Products

Chemical substances derived from natural products, including plants, animals, and microbes, have played a vital role in cancer chemotherapy for the past 30 years [202]. However, developing a new drug is complex, time-consuming, and expensive. It usually takes around 12 years from discovering a new drug in laboratory settings to its application in human diseases. It also involves more than one billion USD of investment in today’s context to develop a new drug from laboratories to clinics [203]. The drug development process in current pharmaceutical settings can be divided into 7 phases, namely: (1) Preclinical research, (2) Investigational New Drug (IND) application, (3) Phase 1 trials, (4) Phase 2 trials, (5) Phase 3 trials, (6) New Drug Application (NDA), and (7) Approval. 

In the case of natural products, the drug discovery process started with rigorous screening procedures using different species of plants on a panel of different cancer cell lines [204]. Different approaches can be used for screening plants to be tested against cancer cell lines. These approaches include the random approach, ethnopharmacology approach [205], traditional system of medicine approach, and zoo-pharmacognosy approach [203]. Once the task of screening potential candidates is over, the following steps include: (1) the screening of biological activity using selective assays, (2) the bioguided fractionation of the identified plant, (3) the isolation and structure elucidation of the active compounds,(4) the evaluation of chemical do-ability, druggability, and patentability, and (5) the decisions based on the safety and biological activity screening [203]. This process is considered the preclinical research phase mentioned earlier. Once this phase is completed, the potential drug needs to go through six remaining phases for drug development before it can reach clinics for human application. 

However, due to the low success rate of finding new chemical entities (NCE) by following this conventional approach, maybe now is the right time for large-scale pharmaceutical organizations to open up the developmental strategies and adopt the nutraceuticals approach. This approach that allows the development of herbal extracts to hit multiple targets as new drugs should be seriously considered. This strategy will not only reduce developmental costs but will also enhance the chances of success in providing effective and safe drugs and minimizing the risk of post-marketing withdrawal. In this study, the active extracts of the plants will be characterized, and the bioactive compounds responsible for the inhibition of prostate cancer cell growth will be used as markers for future standardization of the extract. However, before the standardized active extracts could reach the market for human consumption, additional studies are required to be carried out to investigate the efficacy of the standardized extract on relevant animal models as well as following the regulations set for nutraceutical development in the targeted market. 

One key aspect of the progression from a pharmacological screening of newly discovered anticancer natural products to their clinical use/therapeutic applications is their solubility in water, which seriously affects their bioavailability. When these compounds are administered as herbal extracts, the many phytochemicals present act as co-excipients, thus promoting their solubility [206]. However, when isolated, they are seldom hydrophilic. A classic example is taxol, of which its solubility (less than 0.1 μg/mL in water, 46 mM in ethanol) was compared by its discoverers as being “the same of brick dust” [207]. Therefore, developing strategies to increase the solubility and bioavailability of phytochemicals is critical to their success as medicines. Traditionally, they may include changes in their chemical structures (semi-synthesis) or physical complexation/encapsulation with carriers (cyclodextrin, phospholipids, or other polymers) [208,209,210,211]. State-of-the-art approaches encourage the administration of bioactive molecules/drugs using the oral route to increase patient compliance by conveniently enabling self-administration and flexibility in dosage regimen, thus avoiding sterile conditions for their manufacturing, which reduces production costs. The way forward encompasses nanosuspension and nanoencapsulation technologies [212]. These have been embraced by the scientific community implementing these concepts to deliver phytochemicals in both therapeutical and nutraceutical interventions [213,214].

### 7.2. Research and Development of Nutraceuticals for Prostate Health

The term ‘nutraceuticals’ was first introduced in 1989 by the founder and chairman of the Foundation for Innovation in Medicine (FIM), Dr. Stephen DeFlice. It is a term derived from ‘nutrition’ and ‘pharmaceuticals’ and is defined as any substance that is food or part of a food that provides medical or health benefits, including prevention and treatment of a disease. Recently, the use of nutraceuticals in prostate disease is becoming more popular. Approximately 30% of men diagnosed with prostate disease in North America were reported to use some complementary and alternative medical (CAM) therapy or nutraceutical products [215,216]. Prostate cancer develops over a long period, thus making it suitable for primary, secondary, and tertiary prevention strategies [217]. This is one of the reasons why CAM and nutraceuticals are becoming very popular in promoting prostate health. Besides that, nutraceuticals are not regulated the same way as drugs are, as they are classified according to different food categories. Nutraceuticals’ regulations also vary from country to country and are not as stringent as medicines. This lowers their cost of production and development, so the population can use them appreciably to prevent diseases. Consequently, the global use and trade of nutraceuticals have boomed in recent years, and investment in this sector is attractive to pharmaceuticals companies; for example, GlaxoSmithKline (GSK) has Benecol^®^ for lowering cholesterol, and Bayer markets Iberogast^®^ for functional dyspepsia.

The most popular dietary approaches to promote healthy prostate include popular nutraceuticals such as saw palmettos, *Pygeum africanum*, phytosterols, rye pollen extract (e.g., Cernilton^®^, Graminex LLC, Saginaw), and vitamins and minerals, such as vitamin E and selenium [218]. Despite the lack of stringent regulations associated with the production and development of nutraceuticals, sound research practices are required to provide high-quality products backed by scientific evidence. Therefore, it is common to see scientists involved in pre-clinical research as part of developing nutraceuticals. Following this step, some might continue testing the nutraceuticals’ efficacy on relevant animal models. In contrast, others could directly release the product into the market, providing that the regulations for nutraceutical development in that specific country or region are strictly followed. For example, in the US, nutraceuticals are monitored as ‘dietary supplements’ according to the Dietary Supplement, Health and Education Act (DSHEA) of 1994 [219]. The DSHEA establishes that manufacturers are responsible for the safety evaluation of the product, and if a new ingredient was to be introduced into the product, they must inform the FDA that the new ingredient ‘can reasonably be expected to be safe’ at least 75 days before going to the market. In the European Union (EU), a specific law regulating nutraceuticals does not exist. However, if a claim implies a medicinal benefit for a nutraceutical product, the product needs to comply with the regulatory requirements for medicinal products in respect of safety, efficacy, quality testing, and marketing authorization procedures [219]. EU regulations also state that the beneficial effects of nutraceuticals can only be a ‘health claim’ and not a ‘medicinal claim.’ In Malaysia, no specific law on nutraceuticals exists. However, they are monitored under Malaysia’s Food Act of 1983 and the Food Regulations of 1985, which govern food safety and quality control, including food standards, hygiene, import and export activities, and advertisement and accreditation of laboratories [220]. This law is enforced by the Food Safety and Quality Division (FSQD) of the Ministry of Health (MOH). 

Despite the lack of stringent regulations for nutraceutical development, this should not be an excuse for scientists and manufacturers to ignore good research practices to ensure the production of high-quality nutraceuticals backed by scientific evidence. The food supplements industry may want to play the cards of natural products as modulators of oxidative stress or “killing cancer cells by ROS,” an emerging field in the herbal approach to managing cancer, including prostate cancer [221]. An enormous amount of preclinical and epidemiological data is available to support the potential of phytochemicals to diminish chemotherapeutic-induced toxicity and increase their therapeutic efficiency and survival times in patients [189,222]. Despite the evidence, the clinical implementation is practically inexistent, which is dismal.

## 8. Research and Development of Natural Anticancer Products from Malay Biodiversity

With the global resurgence of interest in natural remedies as a commodity, Malaysia is looking towards capitalizing on its mega biodiversity, which includes the oldest rainforest in the world and an estimated 1200 medicinal plants. A “Herbal Development Office” has been formed to harness this potential under the Ministry of Agriculture (MoA). It outlines the strategic direction, policies, and regulation of research and development clusters that focus on discovery, crop production and agronomy, standardization, product development, toxicology/pre-clinical and clinical studies, and processing technology. 

Since Malaysia is predominantly an agricultural country, it ensures a sufficient supply of raw materials for research and development. With this in mind, and to promote and protect the growth of the local herbal industry, endemic species and varieties of these medicinal plants are under active study to derive high-value herbal supplements and remedies [223]. Accordingly, preference has been given to a subset of 10 traditional plant species with high economic potential (Table 1) [224].

### 8.1. Andrographis paniculata (Burm.f.) Nees

**Figure 1:**
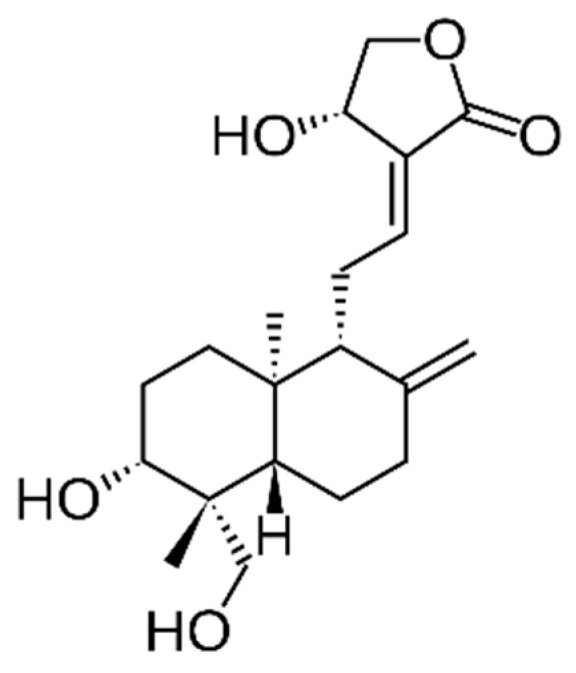
Chemical structure of andrographolide.

The extraction of *A. paniculata* with absolute methanol yields a high andrographolide—a labdane diterpene (Figure 1)—and total phenolic extract with high antiproliferative activity (IC50 ≅ 58 µg/mL) as well as on anti-migration activity on the DU145 cancer cell line. Moreover, it significantly reduces the progression of the cancer cell pre-treated with leptin and adipocyte-conditioned media through cell cycle arrest at the S phase and induction of cell death [225]. Andrographolide has been reported to be the main in vitro bioactive compound of *A. paniculata* against prostate cancer cells [226] without affecting primary prostate epithelial cells [227]. Andrographolide induces apoptosis by modulating the ROS/JNK pathway or ERK-p53 with caspase activation [226,228]. The resulting endoplasmic reticulum stress seems associated with IRE-1 signaling or mitochondrial damage and is accompanied by a marked increase in ROS levels. Similar effects were reported for andrographolide and androgen-independent PC3 cells [226]. It also reduces cell viability and the ability of LNCaP, C4-2b PC3 cells, and DU-145 cells to migrate via modulating CXCL11, CXCR3 and CXCR7 expression as well as Rb, H3, Wee1, and CDC2 phosphorylation [227] and disrupts the IL-6 autocrine loop [229]. This compound affects cell cycle phases in a cell-specific manner: G2/M transition was blocked in LNCaP, C4-2b, and PC3 after treatment, whereas DU-145 cells failed to transit the G1/S phase due to a complex, cell-specific modulation of cyclins A2, B1 and E2 [227]. In vivo studies showed that andrographolide (10 mg/kg *i.v.*, 3 × week) decreased the tumor volume, MMP11 expression, and angiogenesis in an orthotopic xenograft model with androgen-dependent 22RV1 cancer cells. These outcomes were accompanied by increased gene expression in a DNA double-strand break repair, suggesting that andrographolide kills prostate tumor cells by promoting DNA damage [230].

### 8.2. Centella asiatica (L.) Urb

A recent study showing the in vivo effect of *C. asiatica* aqueous extract (100 mg/kg, *per os*, four weeks daily) on a benign prostatic hyperplasia (BPH) rat model induced using testosterone propionate (3 mg/kg *s.c.* daily for four weeks post-castration). The treatment significantly reduced prostate size and its epithelium thickness and androgen receptor signaling-related factors overexpressed by dihydrotestosterone (DHT) treatment in a prostate cell line, such as the expression of the PI3K/Akt pathway and cell proliferation-related factors compared to the untreated group [231]. 

**Figure 2:**
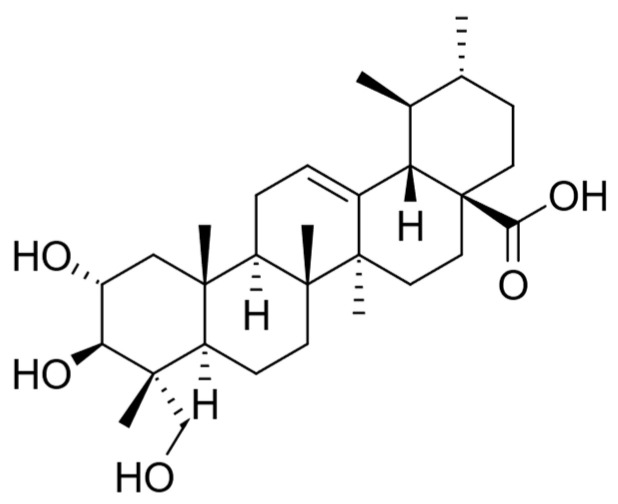
Chemical structure of Asiatic acid, also known as dammarolic acid.

The cytotoxicity of *C. asiatica* extracts and its characteristic compound Asiatic acid—also known as dammarolic acid (Figure 2)—have been reported to induce apoptosis in PC3 cells (20–80 µM, 24 h), with an increase in intracellular ROS and activity of caspase-3 as well as nuclear fragmentation [232]. In addition, this compound has been previously reported to deploy cytotoxicity to other tumor cells. Its mechanisms of action were linked to the inhibition of nuclear factor-κB, p38 mitogen-activated protein kinase, and extracellular signal-regulated kinase, as well as the change in Bcl-2 and caspase family proteins [233,234], modulating the PI3K/protein kinase B (Akt) signaling pathway [235].

### 8.3. Clinacanthus nutans (Burm.f.) Lindau

Despite being popularly claimed to be effective in various cancer treatments by Malaysians [236], there is only a handful of papers providing scientific evidence of its cytotoxic effects in a panel of cancer cell lines that do not include prostate cancer cells. Teoh (2017) reports apoptotic effects of ethanol and ethyl acetate root extracts in MCF-7 cells (IC50 ≅ 30 μg/mL) by suppressing BCL2 with concomitant. A fractionation of a DCM extract of Malaysian origin with similar activity only afforded a fraction with similar IC_50_ containing a heterogenous mix of well-known cytotoxic agents such as epicatechin and 8-gingerol [236]. However, very modest (IC_50_ < 100µg/mL) or not significant effects were observed in HeLa cells [237], A549, CNE1, HepG2 [238] MCF-7, MCF-10A [239], and A549 [240] cancer cells. If there is any base to its widespread use, it may be due to indirect effects on the cancer, such as immunomodulation or other substances arising from the transformation of the plant phytochemicals by the microbiota and/or the human metabolism.

### 8.4. Eurycoma longifolia Jack

*Eurycoma longifolia* (Tongkat Ali) is a popular male tonic in Malaysia and the wider South Asian region. It has been shown to restore serum testosterone levels, thus significantly improving testosterone deficiency syndrome. It appears to be an absolute contraindication in prostate cancer (PCa) patients. However, it has significant positive effects on bone health and patients’ physical condition. In addition, a significant antihyperglycemic effect and cytotoxicity against PCas cells have been the object of a heated debate [241].

This plant contains quassinoid-type diterpenoids, as with many others from the Simaroubaceae family. An extract containing 40% of the total quassinoids found in *E. longifolia* on LNCaP human prostate cancer cell lines showed antiproliferative effects on LNCaP (IC50 ≅ 6 μg/mL) via a G0/G1 phase arrest, which was accompanied by the downregulation of CDK4, CDK2, Cyclin D1, and Cyclin D3, and the upregulation of p21Waf1/Cip1 protein levels. Higher concentrations or a more prolonged treatment duration caused G2M growth arrest, leading to apoptotic cell death. Surprisingly, it shows relatively low toxicity against RWPE-1 human prostate normal cells (IC50 ≅ 60 μg/mL). After the treatment, the quassinoid-rich extract also inhibited 5α-dihydrotestosterone-stimulated growth in LNCaP cells and PSA secretion. Antitumoral effects were also observed in a mouse xenograft model (5 and 10 mg/kg*, i.p*) [242].

### 8.5. Ficus deltoidea L.

*Ficus deltoidea* is a native shrub that belongs to the family of Moraceae. The evergreen small tree or shrub characterizes the plant, and the plant can reach around 5–7 m tall in the wild. This plant species can typically be found in Southeast Asian countries, including Malaysia, Indonesia, and the southern Philippines. It is commonly known as “Mas Cotek” in peninsular Malaysia, and people in east Malaysia commonly refer to this plant as “sempit-sempit” and “agolaran” [243]. This plant plays a vital role in traditional medicine, where different parts of the plant are used for the treatment of several ailments, such as the relief of headaches (fruit part), toothache (fruit part), and sores and wounds (roots and leaves). In addition, women consume the decoction of boiled leaves of *Ficus deltoidea* as an after-birth treatment to contract the uterus and vaginal muscles, besides its treating of the disorders of the menstrual cycle and leucorrhoea [244]. Even though the plant traditionally has many important applications, only a few studies have been conducted to explore its potential pharmacological properties. Abdullah and co-workers [245] reported that the fruit extract of *Ficus deltoidea* showed inhibitory effects against Angiotensin-I converting enzyme (ACE). This finding suggested that the fruit extract has antihypertensive properties. Moreover, tea prepared from *Ficus deltoidea* showed high potential in reducing total cholesterol levels, LDL-cholesterol, and the risk of cardiovascular disease by lowering the antherogenic index (LDL/HDL ratio) and increasing the percentage of the HDL/total cholesterol ratio [246]. This study also finds no sign of cytotoxicity in rats based on a sub-acute toxicity study.

**Figure 3:**
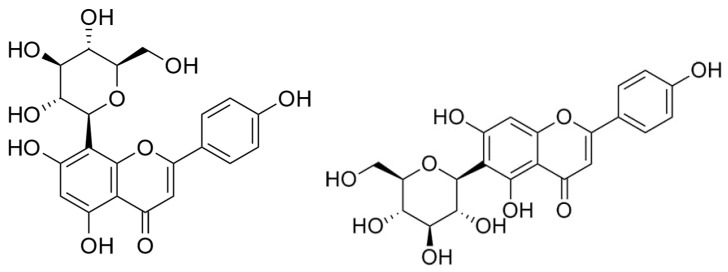
Chemical structure of vitexin (**left**) and isovitexin (**right**).

Flavonoids are abundant in *Ficus deltoidea,* which includes gallocatechin, epigallocatechin, catechin, luteolin-8-C-glucoside, 4-p-coumaroylquinic acid, orientin, vitexin, isovitexin, rutin, quercetin, and naringenin [247]. The presence of these phytochemicals gives its yellow pigmentation, and many studies confirmed that any herbs containing flavonoids could have the ability to act as an anti-inflammatory, anti-allergy, anti-cancer, and anti-microbial agents, so this explains how the plant can protect itself from insects and microorganisms [248,249,250,251]. As mentioned, flavonoids such as epigallocatechin can inhibit the PC3 prostate cancer cell line. Zhou and co-workers [252] have reported that vitexin (Figure 3) showed a cytotoxic effect on breast, ovarian, and prostate cancer cells by inducing apoptosis with the cleavage of the PARP protein, the upregulation of Bax and downregulation of Bcl-2. Rutin, quercetin, and orientin have been reported to have anticancer properties by inducing apoptosis in murine leukemia WEHI-3 cells (rutin) [253], human lung cancer cell line A-549 (quercetin) [254], and human cervical carcinoma cells, HeLa (orientin) [255]. *Ficus* species containing phenanthroindolizidine alkaloids and a series of triterpenoids with C-28 carboxylic acid functional groups are reported to be powerful cytotoxic compounds. Triterpenoids, isolated from the aerial roots of *Ficus microcarpa,* demonstrated cytotoxicity in three human cancer cell lines with IC50 values from 4.0 to 9.4 μM, including HONE-1 nasopharyngeal carcinoma cells, KB oral epidermoid carcinoma cells, and HT29 colorectal carcinoma cells [256,257]. Akhir and co-workers [251] found that both aqueous and ethanolic *Ficus deltoidea* extracts gave IC50 values of 224 and 143 μg/mL, respectively. The aqueous extract caused the detachment of the cancer cell, as observed using the cell viability assay. While DNA fragmentation was not detectable after treatment with the aqueous extract, at 1000 μg/mL of the ethanolic extract, DNA fragmentation occurred around 200 Kbps [251]. Our investigation of the anticancer properties of *Ficus deltoidea* plant extracts and three other varieties of the plants on human prostate cancer cell lines afforded two fractions able to overcome the three main hallmarks of cancer in PC3 cells: apoptosis by activating the intrinsic pathway, inhibition of both migration and invasion by modulating the CXCL12-CXCR4 axis, and inhibiting angiogenesis by modulating VEGF-A expression. Moreover, we reported isovitexin (Figure 3) for the first time as an antiproliferative principle (IC50 = 43 μg/mL) against PC3 cells [258].

### 8.6. Hibiscus sabdariffa L.

The leaves are used as an ingredient in a popular vegetable sour soup to protect against chronic diseases in traditional Thai medicine [259]. These protective properties have been related to its previously shown hypoglycemic, hypolipidemic, and antioxidant effects [260]. The 95% ethanolic extract of these dried leaves is cytotoxic to PC3 cells (IC50 ≅ 9 μg/mL) [259] and induces cell apoptosis. The molecular mechanisms involved in the anticancer activity of *H. sabdariffa* leaf extracts, rich in polyphenols, involve an inhibitory effect on the activity and expressions of matrix metalloproteinase-9 (MMP-9) via the nuclear factor-kappaB (NF-κB)’s inability to bind to DNA. LNCaP cell transfection studies with an Akt1 overexpression vector indicate that this effect may be mediated through the inhibition of the protein kinase B (Akt)/NF-kB/MMP-9 cascade pathway, which results in diminished invasiveness of the prostate cancer cells [260].

**Figure 4:**
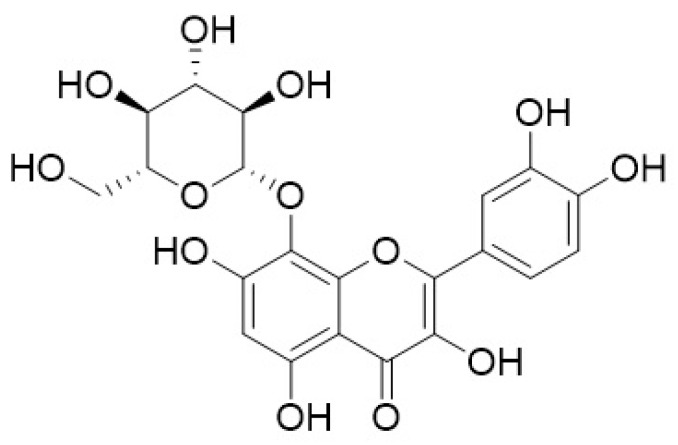
Chemical structure of gossypin.

There are no bioguided studies on the active compounds present in *H. sabdarifa* against prostate cancer cells. However, much research on this plant points towards the flavonoid gossypin (Figure 4), which has significant antioxidant, anti-inflammatory, neuroprotective, anti-cancer, anti-tumor, and anti-diabetic properties [261]. However, recent work expands on understanding other cytotoxic phytochemicals present in *Hibiscus* species such as cadinane-type sesquiterpenoids with apoptotic effects on epithelial breast cancer MDA-MB-231 cells via the inhibition of the PI3Kα pathway [262]. Furthermore, in silico studies are being run on less-explored compounds of *Hibiscus* species. On the one hand, mTOR binding affinities were found for stigmastadienol, lupeol, and taraxasterol acetate—all present in *Hibiscus rosa-sinensis* [263]. On the other hand, the estrogenic activity of polyphenols such as pelargonidin, delphinidin, cyanidin, and hibiscetin has proven to bind to the ER-α subunit more efficiently than cancer drugs such as Tamoxifen and Raloxifene and with a more favorable toxicological profile [264].

### 8.7. Marantodes pumilum (Blume) Kuntze (syn. Labisia pumila (Blume) Mez)

*Marantodes pumilum* (Blume) Kuntze (synonym *Labisia pumila* var *pumila*) belongs to the Myrsinaceae family, locally known as Kacip Fatimah, and is a herb that has been widely used in South East Asian communities for a variety of illnesses and also as health supplements [265]. It is an indigenous medicinal herb of Malaysia and is sometimes referred to locally as Akar Fatimah, Selusoh Fatimah, Tadah Matahari, Rumput Siti Fatimah, Bunga Belangkas Hutan, and Pokok Pinggang. There are three varieties of *Marantodes pumilum*, i.e., *Marantodes pumilum var. alata*, *Marantodes pumilum var. pumila*, and *Marantodes pumilum var. lanceolata*. Each variety has its respective use, and local healers traditionally use var alata and var pumila [266]. They prepare the herb’s extract by boiling the roots, leaves, or whole plant with water, and the extract is taken orally [265,266]. The decoction of the roots is given to pregnant women between one and two months before delivery. This is believed to induce and expedite labor. It has also been widely used, with a long history of use by women in Malaysia to treat post-partum illnesses, assist the contraction of the birth channel, shrink the uterus, improve the menstrual cycle, and expedite weight loss [265]. It was also reported that *Marantodes pumilum* could be used to delay fertility and regain body strength, while other folkloric uses include treatment of flatulence, dysentery, dysmenorrhoea, gonorrhoea, and “sickness in the bones.” Due to its various applications, *Marantodes pumilum* is known as the “queen of plants” of all Malaysian herbs.

**Figure 5:**
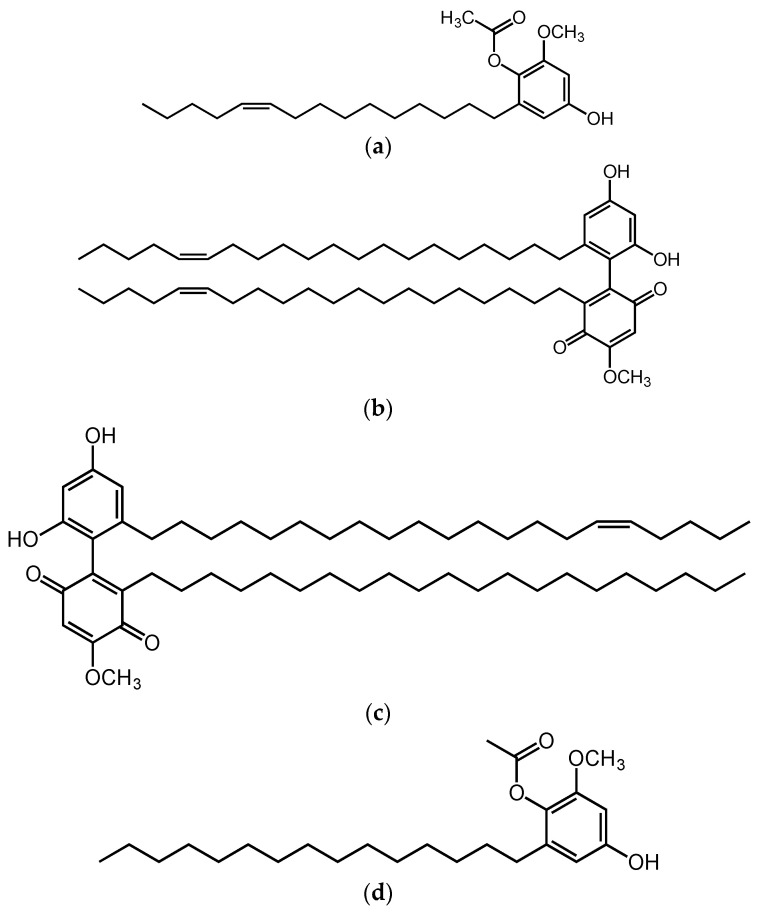
Chemical structure of cytotoxic alkylresorcinols from *M. pumilum*. 1-*O*-methyl-6-acetoxy-5-(pentadec-10*Z*-enyl)resorcinol (**a**), labisiaquinone A (**b**), labisiaquinone B (**c**), and 1-*O*-methyl-6-acetoxy-5-pentadecylresorcinol (**d**).

Al-Mekhlafi and co-workers [267] described Marantodes pumilum extracts’ anti-proliferative properties against different cancer cells. In this study, four compounds isolated from the leaves of *Marantodes pumilum,* namely 1-*O*-methyl-6-acetoxy-5-(pentadic-10*Z*-enyl)resorcinol, labisiaquinone A, labisiaquinone B, and 1-*O*-methyl-6-acetoxy-5-pentadecylresorcinol (Figure 5) showed strong cytotoxicity activity against three different cancer cell lines, PC3 (prostate), HCT116 (colon), and MCF-7 (breast), with low IC50 values (<10 μM). These four compounds also exhibited strong selectivity for PC3 and HCT116 relative to MCF-7. Moreover, the presence of certain phytochemicals such as flavanols (catechin and epigallocatechin), phenolic acids (gallic acid, coumaric acid), flavanols (quercetin, myricetin), and phytoestrogens indicate that the use of this plant could be expanded for other pharmacological applications [268]. Other studies using plants containing similar phytochemical compounds have demonstrated their anticancer properties by addressing some of the hallmarks of cancer. Gallic acid is identified as the major anticancer compound in *Toona sinensis* leaf extract. Studies conducted using this extract have shown that gallic acid is cytotoxic against DU145 prostate cancer cells by generating reactive oxygen species (ROS). It can also block the growth of DU145 cells at G2/M phases by activating Chk1 and Chk2 and inhibiting Cdc25C and Cdc2 [269].

Another phytochemical compound available in *Marantodes pumilum*, epigallocatechin, has been shown to have anticancer properties on another prostate cancer cell line, PC-3. This study has indicated that epigallocatechin could inhibit PC-3 prostate cancer cell proliferation via MEK-independent ERK1/2 activation [270]. 

The regulation of the estrogen receptor alpha (ERalpha) and the estrogen receptor beta (ERbeta) is crucial in prostate cancer prevention [271]. Research has shown that some of the phytochemical compounds in *Marantodes pumilum* extract act as phytoestrogens [272]. Early biological studies on the activity of the phytoestrogen in soy (isoflavones) have demonstrated that phytoestrogen can upregulate the estrogen receptor beta (ERbeta) and downregulate the estrogen receptor alpha (ERalpha), which then lead to the inhibition of prostate cancer cell proliferation [273]. Therefore, it is believed that the phytoestrogen content in *Marantodes pumilum* plant extract could also cause a similar action, which might lead to the death of the cancer cells. Our investigation of the potential of *Marantodes pumilum* (Blume) Kuntze extract in prostate cancer afforded the isolation of 5-henicosene-1-yl-resorcinol and characterized for the first time the detailed mechanism of action of an alkylresorcinol in prostate cancer cells [274].

### 8.8. Morinda citrifolia L.

**Figure 6:**
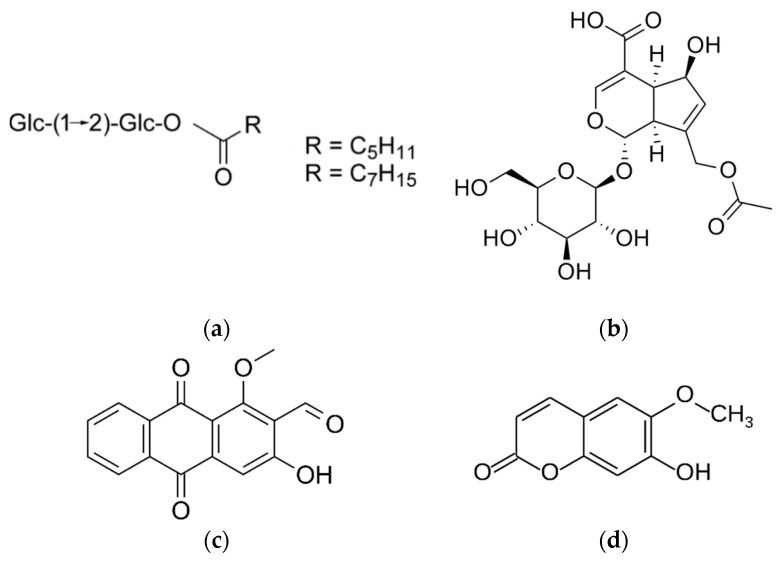
Chemical structure of cytotoxic saccharide fatty acid esters (**a**), asperulosidic acid (**b**), adamnacanthal (**c**), and escopoletin (**d**) present in *M. citrifolia* L.

Noni (the common name of the Hawaiian tree *Morinda citrifolia*) is an extremely popular and fashionable source of food and traditional medicines. In a recent report, traditionally fermented noni juice supplementation in men with very low-risk or low-risk prostate cancer showed stabilizing effects on serum prostate-specific antigen (PSA) levels when administered for one year. In addition, prostate biopsies of the participants showed favorable changes in the expression levels of several genes associated with the androgen pathway, such as FAM13C and KLK2 [275]. However, the active compounds of prostate chemo-preventative effects are not yet known. The presence of saccharide fatty acid esters [276,277], asperulosidic acid [277], and the anthraquinone damnacanthal [278] in the fruits, as well as the rich content of epicatechin and escopoletin in the leaves (Figure 6), may contribute to the cytotoxic effects of its extracts in several types of cancer cells including PC3 [279,280,281,282,283]. A recent review of 51 clinical and preclinical studies showed promising antitumor, antiproliferative, pro-apoptotic, antiangiogenesis, antimigratory, anti-inflammatory, and immunomodulatory activities [275]. However, more studies must be performed to establish if this natural product can be translated into any anticancer intervention.

### 8.9. Orthosiphon aristatus (Blume) Miq.

**Figure 7:**
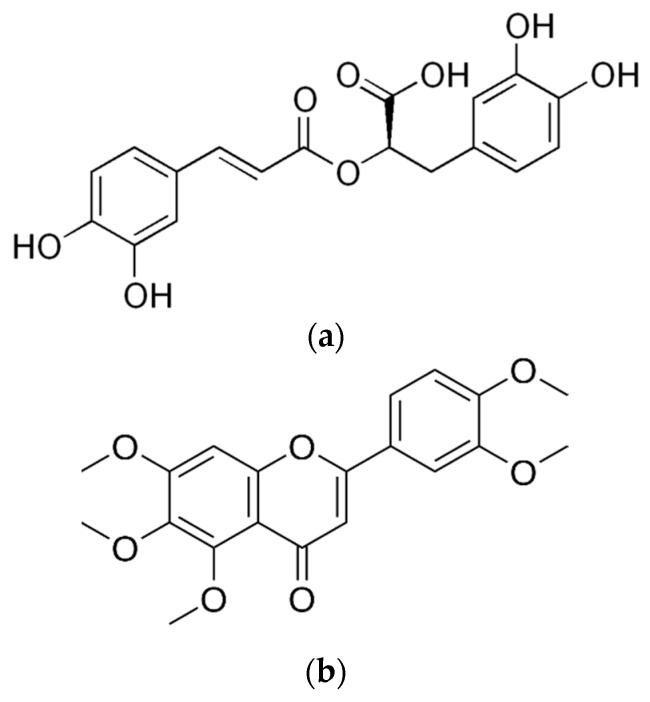
Chemical structure of rosmarinic acid (**a**) and sinensetin (**b**) present in *O. aristatus*.

Cat’s whiskers are consumed as a medicinal tea in Asia for medicinal purposes. Many studies use its synonym, *Orthospihon stamineus*. There has been a recent drive to obtain technologically advanced extracts with cytotoxic effects on prostate cancer cells [281]. One research group optimized ultrasonic-assisted extraction conditions to achieve a maximum yield and rosmarinic acid (Figure 7). Further fractionation of such extract yielded an enriched subextract in rosmarinic acid with an antiproliferative effect against DU145 prostate cancer [282]. Another group used supercritical CO_2_ to obtain an extract containing essential oils, hydrocarbon, fatty acids, esters, and aromatic sesquiterpenes with selective cytotoxicity against prostate cancer PC3 cells (IC50 ≅ 28 µg/mL) via nuclear and mitochondrial apoptotic pathways, as well as inhibitory effects on cell migration even at sub-cytotoxic concentrations [283]. Notably, the extracts cited above show no toxic effect on normal cells.

Sinensetin (Figure 7), also called 3’,4’,5,6,7-pentametoksiflavon, is a plant-derived polymethoxylated flavonoid found in *Orthosiphon aristatus* var. *aristatus* and other citrus fruits. It has been found to possess strong anticancer activities in a panel of cancer cells [281]. However, it is only moderately or weakly active against prostate cancer cells such as SK-MEL5 and DU-145, respectively. These activities increase when the compound is demethylated [284]. There is a need to continue the bioguided isolation of active compounds toward prostate cancer cells.

### 8.10. Phyllanthus niruri L.

*Phyllanthus niruri* is a widespread tropical plant used as a popular remedy for kidney stones, hence one of its common names is “stonebreaker.” It is not to be confounded with *P. emblica,* which yields a popular fruit (Indian gooseberry). Water extracts from Phyllanthus (P*. amarus, P. niruri, P. urinaria, and P. watsonii*) revealed that they lower the expression of Wnt, NFκB, Myc/Max, hypoxia, MAPK/ERK, and MAPK/JNK in PC-3 cells via the pan-Ras, c-Raf, RSK, Elk1, c-Jun, JNK1/2, p38 MAPK, c-myc, DSH, β-catenin, Akt, HIF-1α, GSK3β, NFκB p50 and p52, Bcl-2, Bax, and VEGF. Proteomic-based studies revealed that 72 differentially expressed proteins were involved in tumor cell adhesion, apoptosis, glycogenesis and glycolysis, metastasis, angiogenesis, protein synthesis, and energy metabolism [285]. The same extracts possess mild cytotoxic properties (IC_50_ ≅ 50–300 µg/mL) on MeWo prostate cancer cells without any significant cytotoxicity on normal human skin (CCD-1127Sk) and prostate (RWPE-1) cells. They cause DNA fragmentation and induce caspase-3 and -7 activities with minimal necrotic effects. Furthermore, they induced a Go/G1-phase arrest on PC-3 cells and an S-phase arrest on MeWo cells with an accumulation of cells in the Sub-G1 phase. They significantly inhibited cell adhesion, migration, invasion, and transendothelial migration activities via the inhibition of matrix metalloproteinases -2, -7, -9, and -26 [286]. The authors suggested the presence of polyphenol compounds such as ellagitannins, gallotannins, flavonoids, and phenolic acids as potentially active principles, but no bioguided isolation to substantiate this has been attempted so far.

## 9. Concluding Remarks

The treatment of prostate cancer remains a major clinical and societal challenge. The current chemotherapy drugs armamentarium needs new entries. Natural products may play a significant role in both chemotherapy and combination therapies. The exploitation of natural products as a national marketing objective in Malaysia may provide the scientific drive to add lead compounds to fight prostate cancer. On the one hand, the main active cytotoxic principles against prostate cancer cells have been identified and mechanistically studied in *Andrographis paniculata* (andrographolide)*, Centella asiatica* (Asiatic acid)*, Eurycoma longifolia* (quassinoids)*, Ficus deltoidea* (vitexin, isovitexin)*, Marantodes pumilum (*syn*. Labisia pumila)* (alkylresorcinols)*, Morinda citrifolia* (saccharide fatty acid esters, asperulosidic acid, adamnacanthal, and scopoletin)*,* and *Orthosiphon aristatus* (rosmarinic acid and sinensetin). Commercial products derived from these plants may be developed and implemented in preventative therapy following the model set by saw palmetto. With more intense investment, new anticancer drugs may be derived. On the other hand, C*linacanthus nutans, Hibiscus sabdariffa,* and *Phyllanthus niruri* need further research to unveil their active principles. These offer potential discoveries for those prepared to take on the challenge.

## Figures and Tables

**Table 1:** Traditional plant species with high economic potential according the Malay National Agrofood Policy 2011–2020.

Latin Name	Family	Malay Name	Part Used	Targeted Indications
*Andrographis paniculata* (Burm.f.) Nees	Acanthaceae	Hempedu Bumi	Leaves	Antidiabetic tonic and supplement
*Centella asiatica* (L.) Urb.	Apiaceae	Pegaga	Leaves, stem	Skincare ingredients, herbal drink
*Clinacanthus nutans* (Burm.f.) Lindau	Acanthaceae	Belalai Gajah	Leaves	Herbal drink, anticancer supplement
*Eurycoma longifolia* Jack	Simaroubaceae	Tongkat Ali	Roots	Male tonic, coffee, energy drink
*Ficus deltoidea* Jack	Moraceae	Mas Cotek	Fruit, roots, and leaves	Afterbirth tonic, whitening serum
*Hibiscus sabdariffa* L.	Malvaceae	Roselle	Fruit, leaves	Functional drinks, skincare
*Marantodes pumilum* (Blume) Kuntze (syn. Labisia pumila (Blume) Mez)	Primulaceae	Kacip Fatimah	Leaves and roots	Gynecological, antiaging serum, women’s tonic, and supplement
*Morinda citrifolia* L.	Rubiaceae	Mengkudu	Fruits, leaves, and roots	Herbal drinks, coffee
*Orthosiphon aristatus* (Blume) Miq.	Lamiaceae	Misai Kucing	Stem and leaves	Diuretics, tea, and herbal supplement for kidney disease
*Phyllanthus niruri* L.	Phyllanthaceae.	Dukung Anak	Whole plant	Liver tonic

## Data Availability

Not appliable.

## References

[1] Bray F., Ferlay J., Soerjomataram I., Siegel R.L., Torre L.A., Jemal A. (2018). Global cancer statistics 2018: GLOBOCAN estimates of incidence and mortality worldwide for 36 cancers in 185 countries. CA Cancer J. Clin..

[2] Wang L., Lu B., He M., Wang Y., Wang Z., Du L. (2022). Prostate Cancer Incidence and Mortality: Global Status and Temporal Trends in 89 Countries From 2000 to 2019. Front. Public Health.

[3] Ferlay J., Soerjomataram I., Ervik M., Dikshit R., Eser S., Mathers C., Rebelo M., Parkin D.M., Forman D., Bray F. (2013). GLOBOCAN 2012 v1.0, Cancer Incidence and Mortality Worldwide: IARC CancerBase No. 11.

[4] Cancer Research UK U.K. Prostate Cancer Statistics. http://www.cancerresearchuk.org/health-professional/cancer-statistics/statistics-by-cancer-type/prostate-cancer#heading-Zero.

[5] Institut Kanser Negara (2011). Malaysia National Cancer Registry Report 2007.

[6] Wang M., Takahashi A., Liu F., Ye D., Ding Q., Qin C., Yin C., Zhang Z., Matsuda K., Kubo M. (2015). Large-scale association analysis in Asians identifies new susceptibility loci for prostate cancer. Nat. Commun..

[7] Nelson W.G., De Marzo A.M., Isaacs W.B. (2003). Prostate cancer. N. Engl. J. Med..

[8] Liu M., Hai A., Huang A.T. (1993). Cancer epidemiology in the Far East--contrast with the United States. Oncology.

[9] Wynder E.L., Fujita Y., Harris R.E., Hirayama T., Hiyama T. (1991). Comparative epidemiology of cancer between the united states and japan. A second look. Cancer.

[10] Nelson W.G., Demarzo A.M., Yegnasubramanian S. (2014). The diet as a cause of human prostate cancer. Cancer Treat. Res..

[11] Sugimura T. (2002). Food and cancer. Toxicology.

[12] Coffey D.S. (2001). Similarities of prostate and breast cancer: Evolution, diet, and estrogens. Urology.

[13] Seethalakshmi L., Bala R.S., Malhotra R.K., Austin-Ritchie T., Miller-Graziano C., Menon M., Luber-Narod J. (1996). 17 beta-estradiol induced prostatitis in the rat is an autoimmune disease. J. Urol..

[14] Pescatori S., Berardinelli F., Albanesi J., Ascenzi P., Marino M., Antoccia A., di Masi A., Acconcia F. (2021). A Tale of Ice and Fire: The Dual Role for 17β-Estradiol in Balancing DNA Damage and Genome Integrity. Cancers.

[15] Wu Y., Zhang L., Na R., Xu J., Xiong Z., Zhang N., Dai W., Jiang H., Ding Q. (2015). Plasma genistein and risk of prostate cancer in Chinese population. Int. Urol. Nephrol..

[16] Puangsombat K., Jirapakkul W., Smith J.S. (2011). Inhibitory Activity of Asian Spices on Heterocyclic Amines Formation in Cooked Beef Patties. J. Food Sci..

[17] Morrissey C., Watson R.W. (2003). Phytoestrogens and prostate cancer. Curr. Drug Targets.

[18] Christensen M.J., Quiner T.E., Nakken H.L., Lephart E.D., Eggett D.L., Urie P.M. (2013). Combination effects of dietary soy and methylselenocysteine in a mouse model of prostate cancer. Prostate.

[19] Hwang Y.W., Kim S.Y., Jee S.H., Kim Y.N., Nam C.M. (2009). Soy food consumption and risk of prostate cancer: A meta-analysis of observational studies. Nutr. Cancer.

[20] Sohel M., Sultana H., Sultana T., Mamun A.A., Amin M.N., Hossain M.A., Ali M.C., Aktar S., Sultana A., Rahim Z.B. (2022). Chemotherapeutic Activities of Dietary Phytoestrogens against Prostate Cancer: From Observational to Clinical Studies. Curr. Pharm. Des..

[21] Russo M., Russo G.L., Daglia M., Kasi P.D., Ravi S., Nabavi S.F., Nabavi S.M. (2016). Understanding genistein in cancer: The "good" and the "bad" effects: A review. Food Chem..

[22] Vastag B. (2007). Soy and Prostate Cancer Study Results Mixed. JNCI J. Natl. Cancer Inst..

[23] Nupponen N., Visakorpi T. (1999). Molecular biology of progression of prostate cancer. Eur. Urol..

[24] Hughes C., Murphy A., Martin C., Sheils O., O’Leary J. (2005). Molecular pathology of prostate cancer. J. Clin. Pathol..

[25] Shi X., Sun M., Liu H., Yao Y., Song Y. (2013). Long non-coding RNAs: A new frontier in the study of human diseases. Cancer Lett..

[26] Rubin C. (1998). The Genetic Basis of Human Cancer. Ann. Intern. Med..

[27] Shen M.M., Abate-Shen C. (2010). Molecular genetics of prostate cancer: New prospects for old challenges. Genes Dev..

[28] Fraser M., Sabelnykova V.Y., Yamaguchi T.N., Heisler L.E., Livingstone J., Huang V., Shiah Y.-J., Yousif F., Lin X., Masella A.P. (2017). Genomic hallmarks of localized, non-indolent prostate cancer. Nature.

[29] Cher M.L., Bova G.S., Moore D.H., Small E.J., Carroll P.R., Pin S.S., Epstein J.I., Isaacs W.B., Jensen R.H. (1996). Genetic Alterations in Untreated Metastases and Androgen-independent Prostate Cancer Detected by Comparative Genomic Hybridization and Allelotyping. Cancer Res..

[30] Nupponen N.N., Kakkola L., Koivisto P., Visakorpi T. (1998). Genetic Alterations in Hormone-Refractory Recurrent Prostate Carcinomas. Am. J. Pathol..

[31] Visakorpi T., Kallioniemi A.H., Syvänen A.-C., Hyytinen E.R., Karhu R., Tammela T., Isola J.J., Kallioniemi O.-P. (1995). Genetic Changes in Primary and Recurrent Prostate Cancer by Comparative Genomic Hybridization. Cancer Res..

[32] Taylor B.S., Schultz N., Hieronymus H., Gopalan A., Xiao Y., Carver B.S., Arora V.K., Kaushik P., Cerami E., Reva B. (2010). Integrative genomic profiling of human prostate cancer. Cancer Cell.

[33] Bubendorf L., Kononen J., Koivisto P., Schraml P., Moch H., Gasser T.C., Willi N., Mihatsch M.J., Sauter G., Kallioniemi O.-P. (1999). Survey of Gene Amplifications during Prostate Cancer Progression by High-Throughput Fluorescence in Situ Hybridization on Tissue Microarrays. Cancer Res..

[34] Rickman D.S., Beltran H., Demichelis F., Rubin M.A. (2017). Biology and evolution of poorly differentiated neuroendocrine tumors. Nat. Med..

[35] Min J., Zaslavsky A., Fedele G., McLaughlin S.K., Reczek E.E., De Raedt T., Guney I., Strochlic D.E., MacConaill L.E., Beroukhim R. (2010). An oncogene-tumor suppressor cascade drives metastatic prostate cancer by coordinately activating Ras and nuclear factor-[kappa] B. Nat. Med..

[36] Zeng X., Hu Z., Wang Z., Tao J., Lu T., Yang C., Lee B., Ye Z. (2014). Upregulation of RASGRP3 expression in prostate cancer correlates with aggressive capabilities and predicts biochemical recurrence after radical prostatectomy. Prostate Cancer Prostatic Dis..

[37] Morote J., de Torres I., Caceres C., Vallejo C., Schwartz S., Reventos J. (1999). Prognostic value of immunohistochemical expression of the c-erbB-2 oncoprotein in metastasic prostate cancer. Int. J. Cancer.

[38] Pignon J.-C., Koopmansch B., Nolens G., Delacroix L., Waltregny D., Winkler R. (2009). Androgen receptor controls EGFR and ERBB2 gene expression at different levels in prostate cancer cell lines. Cancer Res..

[39] Yan M., Parker B.A., Schwab R., Kurzrock R. (2014). HER2 aberrations in cancer: Implications for therapy. Cancer Treat. Rev..

[40] Colombel M., Symmans F., Gil S., O’Toole K.M., Chopin D., Benson M., Olsson C.A., Korsmeyer S., Buttyan R. (1993). Detection of the apoptosis-suppressing oncoprotein bc1-2 in hormone-refractory human prostate cancers. Am. J. Pathol..

[41] Krajewska M., Krajewski S., Epstein J.I., Shabaik A., Sauvageot J., Song K., Kitada S., Reed J.C. (1996). Immunohistochemical analysis of bcl-2, bax, bcl-X, and mcl-1 expression in prostate cancers. Am. J. Pathol..

[42] McDonnell T.J., Troncoso P., Brisbay S.M., Logothetis C., Chung L.W.K., Hsieh J.-T., Tu S.-M., Campbell M.L. (1992). Expression of the Protooncogene bcl-2 in the Prostate and Its Association with Emergence of Androgen-independent Prostate Cancer. Cancer Res..

[43] Pienta K.J., Bradley D. (2006). Mechanisms underlying the development of androgen-independent prostate cancer. Clin. Cancer Res..

[44] Delbridge A.R.D., Grabow S., Strasser A., Vaux D.L. (2016). Thirty years of BCL-2: Translating cell death discoveries into novel cancer therapies. Nat. Rev. Cancer.

[45] Velcheti V., Karnik S., Bardot S.F., Prakash O. (2008). Pathogenesis of Prostate Cancer: Lessons from Basic Research. Ochsner J..

[46] Edwards J., Bartlett J. (2005). The androgen receptor and signal-transduction pathways in hormone-refractory prostate cancer. Part 2: Androgen-receptor cofactors and bypass pathways. BJU Int..

[47] Chmelar R., Buchanan G., Need E.F., Tilley W., Greenberg N.M. (2007). Androgen receptor coregulators and their involvement in the development and progression of prostate cancer. Int. J. Cancer.

[48] Denmeade S.R., Lin X.S., Isaacs J.T. (1996). Role of programmed (apoptotic) cell death during the progression and therapy for prostate cancer. Prostate.

[49] Tan M.H.E., Li J., Xu H.E., Melcher K., Yong E.-l. (2015). Androgen receptor: Structure, role in prostate cancer and drug discovery. Acta Pharmacol. Sin..

[50] Scher H.I., Sawyers C.L. (2005). Biology of progressive, castration-resistant prostate cancer: Directed therapies targeting the androgen-receptor signaling axis. J. Clin. Oncol..

[51] Roudier M.P., Morrissey C., True L.D., Higano C.S., Vessella R.L., Ott S.M. (2008). Histopathological assessment of prostate cancer bone osteoblastic metastases. J. Urol..

[52] Rubens R. (1998). Bone metastases—The clinical problem. Eur. J. Cancer.

[53] Fizazi K., Carducci M., Smith M., Damião R., Brown J., Karsh L., Milecki P., Shore N., Rader M., Wang H. (2011). Denosumab versus zoledronic acid for treatment of bone metastases in men with castration-resistant prostate cancer: A randomised, double-blind study. Lancet.

[54] Geldof A. (1996). Models for cancer skeletal metastasis: A reappraisal of Batson’s plexus. Anticancer Res..

[55] Diel I. (1994). Historical remarks on metastasis and metastatic bone disease. Metastatic Bone Disease.

[56] Kim C.H., Broxmeyer H.E. (1999). SLC/exodus2/6Ckine/TCA4 induces chemotaxis of hematopoietic progenitor cells: Differential activity of ligands of CCR7, CXCR3, or CXCR4 in chemotaxis vs. suppression of progenitor proliferation. J. Leukoc. Biol..

[57] Aiuti A., Tavian M., Cipponi A., Ficara F., Zappone E., Hoxie J., Peault B., Bordignon C. (1999). Expression of CXCR4, the receptor for stromal cell-derived factor-1 on fetal and adult human lymphohematopoietic progenitors. Eur. J. Immunol..

[58] Taichman R.S., Cooper C., Keller E.T., Pienta K.J., Taichman N.S., McCauley L.K. (2002). Use of the Stromal Cell-derived Factor-1/CXCR4 Pathway in Prostate Cancer Metastasis to Bone. Cancer Res..

[59] Sun Y.X., Wang J., Shelburne C.E., Lopatin D.E., Chinnaiyan A.M., Rubin M.A., Pienta K.J., Taichman R.S. (2003). Expression of CXCR4 and CXCL12 (SDF-1) in human prostate cancers (PCa) in vivo. J. Cell. Biochem..

[60] Müller A., Homey B., Soto H., Ge N., Catron D., Buchanan M.E., McClanahan T., Murphy E., Yuan W., Wagner S.N. (2001). Involvement of chemokine receptors in breast cancer metastasis. Nature.

[61] Morton R.A., Ewing C.M., Nagafuchi A., Tsukita S., Isaacs W.B. (1993). Reduction of E-Cadherin Levels and Deletion of the α-Catenin Gene in Human Prostate Cancer Cells. Cancer Res..

[62] Umbas R., Schalken J.A., Aalders T.W., Carter B.S., Karthaus H.F.M., Schaafsma H.K., Debruyne F.M.J., Isaacs W.B. (1992). Expression of the Cellular Adhesion Molecule E-Cadherin Is Reduced or Absent in High-Grade Prostate Cancer. Cancer Res..

[63] Mashimo T., Watabe M., Hirota S., Hosobe S., Miura K., Tegtmeyer P.J., Rinker-Shaeffer C.W., Watabe K. (1998). The expression of the KAI1 gene, a tumor metastasis suppressor, is directly activated by p53. Proc. Natl. Acad. Sci. USA.

[64] Hida T., Yatabe Y., Achiwa H., Muramatsu H., Kozaki K.-i., Nakamura S., Ogawa M., Mitsudomi T., Sugiura T., Takahashi T. (1998). Increased expression of cyclooxygenase 2 occurs frequently in human lung cancers, specifically in adenocarcinomas. Cancer Res..

[65] Tucker O.N., Dannenberg A.J., Yang E.K., Zhang F., Teng L., Daly J.M., Soslow R.A., Masferrer J.L., Woerner B.M., Koki A.T. (1999). Cyclooxygenase-2 expression is up-regulated in human pancreatic cancer. Cancer Res..

[66] Aggarwal B.B., Shishodia S. (2006). Molecular targets of dietary agents for prevention and therapy of cancer. Biochem. Pharmacol..

[67] Wolfesberger B., Walter I., Hoelzl C., Thalhammer J.G., Egerbacher M. (2006). Antineoplastic effect of the cyclooxygenase inhibitor meloxicam on canine osteosarcoma cells. Res. Vet. Sci..

[68] Eberhart C.E., Coffey R.J., Radhika A., Giardiello F.M., Ferrenbach S., Dubois R.N. (1994). Up-regulation of cyclooxygenase 2 gene expression in human colorectal adenomas and adenocarcinomas. Gastroenterology.

[69] Koga H., Sakisaka S., Ohishi M., Kawaguchi T., Taniguchi E., Sasatomi K., Harada M., Kusaba T., Tanaka M., Kimura R. (1999). Expression of cyclooxygenase-2 in human hepatocellular carcinoma: Relevance to tumor dedifferentiation. Hepatology.

[70] Hwang D., Byrne J., Scollard D., Levine E. (1998). Expression of cyclooxygenase-1 and cyclooxygenase-2 in human breast cancer. J. Natl. Cancer Inst..

[71] Mohammed S.I., Knapp D.W., Bostwick D.G., Foster R.S., Khan K.N.M., Masferrer J.L., Woerner B.M., Snyder P.W., Koki A.T. (1999). Expression of cyclooxygenase-2 (COX-2) in human invasive transitional cell carcinoma (TCC) of the urinary bladder. Cancer Res..

[72] Ghosh J., Myers C.E. (1997). Arachidonic Acid Stimulates Prostate Cancer Cell Growth: Critical Role of 5-Lipoxygenase. Biochem. Biophys. Res. Commun..

[73] Yang P., Cartwright C.A., Li J.I.N., Wen S., Prokhorova I.N., Shureiqi I., Troncoso P., Navone N.M., Newman R.A., Kim J. (2012). Arachidonic acid metabolism in human prostate cancer. Int. J. Oncol..

[74] Werz O., Steinhilber D. (2006). Therapeutic options for 5-lipoxygenase inhibitors. Pharmacol. Ther..

[75] Rådmark O., Werz O., Steinhilber D., Samuelsson B. (2007). 5-Lipoxygenase: Regulation of expression and enzyme activity. Trends Biochem. Sci..

[76] Fürstenberger G., Krieg P., Müller-Decker K., Habenicht A.J.R. (2006). What are cyclooxygenases and lipoxygenases doing in the driver’s seat of carcinogenesis?. Int. J. Cancer.

[77] Ghosh J. (2008). Targeting 5-lipoxygenase for prevention and treatment of cancer. Curr. Enzym. Inhib..

[78] Holmgren L., O’Reilly M.S., Folkman J. (1995). Dormancy of micrometastases: Balanced proliferation and apoptosis in the presence of angiogenesis suppression. Nat. Med..

[79] Carmeliet P. (2003). Angiogenesis in health and disease. Nat. Med..

[80] Li R., Younes M., Wheeler T.M., Scardino P., Ohori M., Frolov A., Ayala G. (2004). Expression of vascular endothelial growth factor receptor-3 (VEGFR-3) in human prostate. Prostate.

[81] Ferrara N., Gerber H.-P., LeCouter J. (2003). The biology of VEGF and its receptors. Nat. Med..

[82] Friesel R.E., Maciag T. (1995). Molecular mechanisms of angiogenesis: Fibroblast growth factor signal transduction. FASEB J..

[83] Battegay E.J., Rupp J., Iruela-Arispe L., Sage E.H., Pech M. (1994). PDGF-BB modulates endothelial proliferation and angiogenesis in vitro via PDGF beta-receptors. J. Cell Biol..

[84] Wang B., Hendricks D.T., Wamunyokoli F., Parker M.I. (2006). A Growth-Related Oncogene/CXC Chemokine Receptor 2 Autocrine Loop Contributes to Cellular Proliferation in Esophageal Cancer. Cancer Res..

[85] Sankar S., Mahooti-Brooks N., Bensen L., McCarthy T.L., Centrella M., Madri J.A. (1996). Modulation of transforming growth factor beta receptor levels on microvascular endothelial cells during in vitro angiogenesis. J. Clin. Investig..

[86] Egeblad M., Werb Z. (2002). New functions for the matrix metalloproteinases in cancer progression. Nat. Rev. Cancer.

[87] He Y., Kozaki K.-i., Karpanen T., Koshikawa K., Yla-Herttuala S., Takahashi T., Alitalo K. (2002). Suppression of Tumor Lymphangiogenesis and Lymph Node Metastasis by Blocking Vascular Endothelial Growth Factor Receptor 3 Signaling. J. Natl. Cancer Inst..

[88] Murphy G., Ragde H., Kenny G., Barren R., Erickson S., Tjoa B., Boynton A., Holmes E., Gilbaugh J., Douglas T. (1995). Comparison of prostate specific membrane antigen, and prostate specific antigen levels in prostatic cancer patients. Anticancer Res..

[89] Chang S.S., Heston W.D. (2002). The clinical role of prostate-specific membrane antigen (PSMA). Urol. Oncol..

[90] Conway R.E., Petrovic N., Li Z., Heston W., Wu D., Shapiro L.H. (2006). Prostate-specific membrane antigen regulates angiogenesis by modulating integrin signal transduction. Mol. Cell Biol..

[91] Gao Y., Zheng H., Li L., Feng M., Chen X., Hao B., Lv Z., Zhou X., Cao Y. (2021). Prostate-Specific Membrane Antigen (PSMA) Promotes Angiogenesis of Glioblastoma Through Interacting With ITGB4 and Regulating NF-κB Signaling Pathway. Front. Cell Dev. Biol..

[92] Bradbury R., Jiang W.G., Cui Y.-X. (2015). The clinical and therapeutic uses of MDM2 and PSMA and their potential interaction in aggressive cancers. Biomark. Med..

[93] Watanabe R., Maekawa M., Kiyoi T., Kurata M., Miura N., Kikugawa T., Higashiyama S., Saika T. (2021). PSMA-positive membranes secreted from prostate cancer cells have potency to transform vascular endothelial cells into an angiogenic state. Prostate.

[94] Machado C.M.L., Skubal M., Haedicke K., Silva F.P., Stater E.P., de Oliveira Silva T.L.A., Costa E.T., Masotti C., Otake A.H., Andrade L.N.S. (2022). PSMA-bearing extracellular vesicles secreted from prostate cancer convert the microenvironment to a tumor-supporting, pro-angiogenic state. bioRxiv.

[95] Brawley O.W. (2002). The Potential for Prostate Cancer Chemoprevention. Rev. Urol..

[96] Sporn M.B., Newton D.L. (1979). Chemoprevention of cancer with retinoids. Federation Proceedings.

[97] Kelloff G.J., Lieberman R., Steele V.E., Boone C.W., Lubet R.A., Kopelovitch L., Malone W.A., Crowell J.A., Sigman C.C. (1999). Chemoprevention of prostate cancer: Concepts and strategies. Eur. Urol..

[98] Sandhu G.S., Nepple K.G., Tanagho Y.S., Andriole G.L. (2013). Prostate cancer chemoprevention. Seminars in Oncology.

[99] Gonzalgo M.L., Isaacs W.B. (2003). Molecular pathways to prostate cancer. J. Urol..

[100] Epstein J.I., Herawi M. (2006). Prostate needle biopsies containing prostatic intraepithelial neoplasia or atypical foci suspicious for carcinoma: Implications for patient care. J. Urol..

[101] Stamatiou K., Alevizos A., Agapitos E., Sofras F. (2006). Incidence of impalpable carcinoma of the prostate and of non-malignant and precarcinomatous lesions in Greek male population: An autopsy study. Prostate.

[102] Hong W.K., Lippman S.M. (1994). Cancer chemoprevention. J. Natl. Cancer Inst. Monogr..

[103] Walsh P.C. (2010). Chemoprevention of prostate cancer. N. Engl. J. Med..

[104] Tsukamoto S., Akaza H., Onozawa M., Shirai T., Ideyama Y. (1998). A five-alpha reductase inhibitor or an antiandrogen prevents the progression of microscopic prostate carcinoma to macroscopic carcinoma in rats. Cancer.

[105] Roehrborn C.G., Boyle P., Bergner D., Gray T., Gittelman M., Shown T., Melman A., Bracken R.B., deVere White R., Taylor A. (1999). Serum prostate-specific antigen and prostate volume predict long-term changes in symptoms and flow rate: Results of a four-year, randomized trial comparing finasteride versus placebo. Urology.

[106] Stoner E. (1994). Three-year safety and efficacy data on the use of finasteride in the treatment of benign prostatic hyperplasia. Urology.

[107] Bramson H.N., Hermann D., Batchelor K.W., Lee F.W., James M.K., Frye S.V. (1997). Unique preclinical characteristics of GG745, a potent dual inhibitor of 5AR. J. Pharmacol. Exp. Ther..

[108] Andriole G.L., Bostwick D.G., Brawley O.W., Gomella L.G., Marberger M., Montorsi F., Pettaway C.A., Tammela T.L., Teloken C., Tindall D.J. (2010). Effect of Dutasteride on the Risk of Prostate Cancer. N. Engl. J. Med..

[109] Lu-Yao G.L., Albertsen P.C., Moore D.F., Shih W., Lin Y., DiPaola R.S., Barry M.J., Zietman A., O’Leary M., Walker-Corkery E. (2009). Outcomes of Localized Prostate Cancer Following Conservative Management. JAMA J. Am. Med. Assoc..

[110] Mongiat-Artus P., Peyromaure M., Richaud P., Droz J.P., Rainfray M., Jeandel C., Rebillard X., Moreau J.L., Davin J.L., Salomon L. (2009). Recommandations pour la prise en charge du cancer de la prostate chez l’homme âgé: Un travail du comité de cancérologie de l’association française d’urologie. Progrès En Urol..

[111] Picard J.C., Golshayan A.-R., Marshall D.T., Opfermann K.J., Keane T.E. (2012). The multi-disciplinary management of high-risk prostate cancer. Urol. Oncol. Semin. Orig. Investig..

[112] Pound C. (1999). Natural History of Progression After PSA Elevation Following Radical Prostatectomy. JAMA J. Am. Med. Assoc..

[113] Uchio E. (2010). Impact of Biochemical Recurrence in Prostate Cancer Among US Veterans. Arch. Intern. Med..

[114] Antonarakis E., Trock B., Feng Z., Humphreys E., Carducci M., Partin A., Walsh P., Eisenberger M. (2009). The natural history of metastatic progression in men with PSA-recurrent prostate cancer after radical prostatectomy: 25-year follow-up. J. Clin. Oncol..

[115] D’Amico A., Cote K., Loffredo M., Renshaw A., Schultz D. (2002). Determinants of prostate cancer-specific survival after radiation therapy for patients with clinically localized prostate cancer. J. Clin. Oncol..

[116] Paller C.J., Antonarakis E.S. (2011). Cabazitaxel: A novel second-line treatment for metastatic castration-resistant prostate cancer. Drug Des. Dev. Ther..

[117] Tannock I.F., Osoba D., Stockler M.R., Ernst D.S., Neville A.J., Moore M.J., Armitage G.R., Wilson J.J., Venner P.M., Coppin C.M. (1996). Chemotherapy with mitoxantrone plus prednisone or prednisone alone for symptomatic hormone-resistant prostate cancer: A Canadian randomized trial with palliative end points. J. Clin. Oncol..

[118] Petrylak D., Petrylak C., Tangen M.H.A., Hussain P., Lara J., Jones M., Taplin P., Burch D., Berry C., Moinpour M. (2004). Docetaxel and Estramustine Compared with Mitoxantrone and Prednisone for Advanced Refractory Prostate Cancer. N. Engl. J. Med..

[119] Fiorica F., Buttigliero C., Grigolato D., Muraro M., Turco F., Munoz F., Tucci M. (2022). Addition of New Androgen Receptor Pathway Inhibitors to Docetaxel and Androgen Deprivation Therapy in Metastatic Hormone-Sensitive Prostate Cancer: A Systematic Review and Metanalysis. Curr. Oncol..

[120] D’Aniello C., Cavaliere C., Foglia C., Facchini S., Uricchio F., Balsamo R., Franzese E., De Falco S., Izzo M., Laterza M. (2022). Management of systemic prostate cancer: Current algorithm from castration sensitive to castration resistant setting. Eur. Rev. Med. Pharmacol. Sci..

[121] Pozas J., Álvarez Rodríguez S., Fernández V.A., Burgos J., Santoni M., Manneh Kopp R., Molina-Cerrillo J., Alonso-Gordoa T. (2022). Androgen Receptor Signaling Inhibition in Advanced Castration Resistance Prostate Cancer: What Is Expected for the Near Future?. Cancers.

[122] Chen K., O’Brien J., McVey A., Jenjitranant P., Kelly B.D., Kasivisvanathan V., Lawrentschuk N., Murphy D.G., Azad A.A. (2022). Combination treatment in metastatic prostate cancer: Is the bar too high or have we fallen short?. Nat. Rev. Urol..

[123] Higano C.S., Hafron J. (2022). Adherence with Oral Anticancer Therapies: Clinical Trial vs Real-world Experiences with a Focus on Prostate Cancer. J. Urol..

[124] Klose P., Werner M., Saha F., Voiß P. (2022). Mind-body medicine in integrative uro-oncology: Studies and areas of application. Urologie.

[125] Kumar R., Sena L.A., Denmeade S.R., Kachhap S. (2022). The testosterone paradox of advanced prostate cancer: Mechanistic insights and clinical implications. Nat. Rev. Urol..

[126] Hanahan D., Weinberg R.A. (2000). The hallmarks of cancer. Cell.

[127] Hanahan D., Weinberg R.A. (2011). Hallmarks of cancer: The next generation. Cell.

[128] Blandin A.-F., Renner G., Lehmann M., Lelong-Rebel I., Martin S., Dontenwill M. (2015). β1 Integrins as Therapeutic Targets to Disrupt Hallmarks of Cancer. Front. Pharmacol..

[129] Vaux D.L., Korsmeyer S.J. (1999). Cell death in development. Cell.

[130] Martinez-Ruiz G., Maldonado V., Ceballos-Cancino G., Grajeda J.P.R., Melendez-Zajgla J. (2008). Role of Smac/DIABLO in cancer progression. J. Exp. Clin. Cancer Res. CR.

[131] Walczak H., Krammer P.H. (2000). The CD95 (APO-1/Fas) and the TRAIL (APO-2L) Apoptosis Systems. Exp. Cell Res..

[132] Fulda S., Debatin K. (2006). Extrinsic versus intrinsic apoptosis pathways in anticancer chemotherapy. Oncogene.

[133] Green D.R., Kroemer G. (2004). The Pathophysiology of Mitochondrial Cell Death. Science.

[134] Candé C., Cecconi F., Dessen P., Kroemer G. (2002). Apoptosis-inducing factor (AIF): Key to the conserved caspase-independent pathways of cell death?. J. Cell Sci..

[135] Saelens X., Festjens N., Walle L.V., Van Gurp M., van Loo G., Vandenabeele P. (2004). Toxic proteins released from mitochondria in cell death. Oncogene.

[136] Du C., Fang M., Li Y., Li L., Wang X. (2000). Smac, a mitochondrial protein that promotes cytochrome c–dependent caspase activation by eliminating IAP inhibition. Cell.

[137] Verhagen A.M., Ekert P.G., Pakusch M., Silke J., Connolly L.M., Reid G.E., Moritz R.L., Simpson R.J., Vaux D.L. (2000). Identification of DIABLO, a mammalian protein that promotes apoptosis by binding to and antagonizing IAP proteins. Cell.

[138] Jia L., Patwari Y., Kelsey S.M., Srinivasula S.M., Agrawal S.G., Alnemri E.S., Newland A.C. (2003). Role of Smac in human leukaemic cell apoptosis and proliferation. Oncogene.

[139] Deveraux Q.L., Roy N., Stennicke H.R., Van Arsdale T., Zhou Q., Srinivasula S.M., Alnemri E.S., Salvesen G.S., Reed J.C. (1998). IAPs block apoptotic events induced by caspase-8 and cytochrome c by direct inhibition of distinct caspases. EMBO J..

[140] Srinivasula S.M., Hegde R., Saleh A., Datta P., Shiozaki E., Chai J., Lee R.-A., Robbins P.D., Fernandes-Alnemri T., Shi Y. (2001). A conserved XIAP-interaction motif in caspase-9 and Smac/DIABLO regulates caspase activity and apoptosis. Nature.

[141] Carson J.P., Behnam M., Sutton J.N., Du C., Wang X., Hunt D.F., Weber M.J., Kulik G. (2002). Smac is required for cytochrome c-induced apoptosis in prostate cancer LNCaP cells. Cancer Res..

[142] Guo F., Nimmanapalli R., Paranawithana S., Wittman S., Griffin D., Bali P., O’Bryan E., Fumero C., Wang H.G., Bhalla K. (2002). Ectopic overexpression of second mitochondria-derived activator of caspases (Smac/DIABLO) or cotreatment with N-terminus of Smac/DIABLO peptide potentiates epothilone B derivative–(BMS 247550) and Apo-2L/TRAIL–induced apoptosis. Blood.

[143] Fulda S., Wick W., Weller M., Debatin K.-M. (2002). Smac agonists sensitize for Apo2L/TRAIL-or anticancer drug-induced apoptosis and induce regression of malignant glioma in vivo. Nat. Med..

[144] Perimenis P., Galaris A., Voulgari A., Prassa M., Pintzas A. (2016). IAP antagonists Birinapant and AT-406 efficiently synergise with either TRAIL, BRAF, or BCL-2 inhibitors to sensitise BRAFV600E colorectal tumour cells to apoptosis. BMC Cancer.

[145] Chinni S.R., Sivalogan S., Dong Z., Deng X., Bonfil R.D., Cher M.L. (2006). CXCL12/CXCR4 signaling activates Akt-1 and MMP-9 expression in prostate cancer cells: The role of bone microenvironment-associated CXCL12. Prostate.

[146] Singareddy R., Semaan L., Conley-LaComb M.K., John J.S., Powell K., Iyer M., Smith D., Heilbrun L.K., Shi D., Sakr W. (2013). Transcriptional regulation of CXCR4 in prostate cancer: Significance of TMPRSS2-ERG fusions. Mol. Cancer Res..

[147] Conley-LaComb M.K., Saliganan A., Kandagatla P., Chen Y.Q., Cher M.L., Chinni S.R. (2013). PTEN loss mediated Akt activation promotes prostate tumor growth and metastasis via CXCL12/CXCR4 signaling. Mol. Cancer.

[148] Sun X., Cheng G., Hao M., Zheng J., Zhou X., Zhang J., Taichman R.S., Pienta K.J., Wang J. (2010). CXCL12/CXCR4/CXCR7 Chemokine Axis and Cancer Progression. Cancer Metastasis Rev..

[149] Vindrieux D., Escobar P., Lazennec G. (2009). Emerging roles of chemokines in prostate cancer. Endocr.-Relat. Cancer.

[150] Thelen M. (2001). Dancing to the tune of chemokines. Nat. Immunol..

[151] Gillette J.M., Larochelle A., Dunbar C.E., Lippincott-Schwartz J. (2009). Intercellular transfer to signalling endosomes regulates an ex vivo bone marrow niche. Nat. Cell Biol..

[152] Kyriakou C., Rabin N., Pizzey A., Nathwani A., Yong K. (2008). Factors that influence short-term homing of human bone marrow-derived mesenchymal stem cells in a xenogeneic animal model. Haematologica.

[153] Sun Y.X., Schneider A., Jung Y., Wang J., Dai J., Wang J., Cook K., Osman N.I., Koh-Paige A.J., Shim H. (2005). Skeletal localization and neutralization of the SDF-1 (CXCL12)/CXCR4 axis blocks prostate cancer metastasis and growth in osseous sites in vivo. J. Bone Miner. Res..

[154] Engl T., Relja B., Marian D., Blumenberg C., Müller I., Beecken W.-D., Jones J., Ringel E.M., Bereiter-Hahn J., Jonas D. (2006). CXCR4 chemokine receptor mediates prostate tumor cell adhesion through α5 and β3 integrins. Neoplasia.

[155] Lee J.Y., Kang D.H., Chung D.Y., Kwon J.K., Lee H., Cho N.H., Choi Y.D., Hong S.J., Cho K.S. (2014). Meta-analysis of the relationship between CXCR4 expression and metastasis in prostate cancer. World J. Men’s Health.

[156] Folkman J. (1990). What is the evidence that tumors are angiogenesis dependent?. CancerSpectrum Knowl. Environ..

[157] Ferrara N., Hillan K.J., Gerber H.-P., Novotny W. (2004). Discovery and development of bevacizumab, an anti-VEGF antibody for treating cancer. Nat. Rev. Drug Discov..

[158] Ide A.G., Baker N.H., Warren S.L. (1939). Vascularization of the Brown Pearce rabbit epithelioma transplant as seen in the transparent ear chamber. Am. J. Roentgenol..

[159] Algire G.H., Chalkley H.W., Legallais F.Y., Park H.D. (1945). Vascular reactions of normal and malignant tissues in vivo. I. Vascular reactions of mice to wounds and to normal and neoplastic transplants. J. Natl. Cancer Inst..

[160] Algire G.H. (1943). An adaptation of the transparent-chamber technique to the mouse. J. Natl. Cancer Inst..

[161] Folkman J. (1971). Tumor angiogenesis: Therapeutic implications. N. Engl. J. Med..

[162] Folkman J., Klagsbrun M. (1987). Angiogenic factors. Science.

[163] Klagsbrun M., D’Amore P.A. (1991). Regulators of angiogenesis. Annu. Rev. Physiol..

[164] Senger D.R., Galli S.J., Dvorak A.M., Perruzzi C.A., Harvey V.S., Dvorak H.F. (1983). Tumor cells secrete a vascular permeability factor that promotes accumulation of ascites fluid. Science.

[165] Ferrara N., Henzel W.J. (1989). Pituitary follicular cells secrete a novel heparin-binding growth factor specific for vascular endothelial cells. Biochem. Biophys. Res. Commun..

[166] Carmeliet P. (2005). VEGF as a Key Mediator of Angiogenesis in Cancer. Oncology.

[167] Hicklin D.J., Ellis L.M. (2005). Role of the Vascular Endothelial Growth Factor Pathway in Tumor Growth and Angiogenesis. J. Clin. Oncol..

[168] Ferrara N., Carver-Moore K., Chen H., Dowd M. (1996). Heterozygous embryonic lethality induced by targeted inactivation of the VEGF gene. Nature.

[169] Ferrara N. (1999). Molecular and biological properties of vascular endothelial growth factor. J. Mol. Med..

[170] Ferrara N. (2001). Role of vascular endothelial growth factor in regulation of physiological angiogenesis. Am. J. Physiol.-Cell Physiol..

[171] Ferrara N., Davis-Smyth T. (1997). The biology of vascular endothelial growth factor. Endocr. Rev..

[172] Shibuya M., Yamaguchi S., Yamane A., Ikeda T., Tojo A., Matsushime H., Sato M. (1990). Nucleotide sequence and expression of a novel human receptor-type tyrosine kinase gene (flt) closely related to the fms family. Oncogene.

[173] Terman B.I., Dougher-Vermazen M., Carrion M.E., Dimitrov D., Armellino D.C., Gospodarowicz D., Böhlen P. (1992). Identification of the KDR tyrosine kinase as a receptor for vascular endothelial cell growth factor. Biochem. Biophys. Res. Commun..

[174] Paavonen K., Puolakkainen P., Jussila L., Jahkola T., Alitalo K. (2000). Vascular endothelial growth factor receptor-3 in lymphangiogenesis in wound healing. Am. J. Pathol..

[175] Kaipainen A., Korhonen J., Mustonen T., Van Hinsbergh V., Fang G.-H., Dumont D., Breitman M., Alitalo K. (1995). Expression of the fms-like tyrosine kinase 4 gene becomes restricted to lymphatic endothelium during development. Proc. Natl. Acad. Sci. USA.

[176] Cao Y., Chen H., Zhou L., Chiang M.-K., Anand-Apte B., Weatherbee J.A., Wang Y., Fang F., Flanagan J.G., Tsang M.L.-S. (1996). Heterodimers of Placenta growth factor/vascular endothelial growth factor endothelial activity, tumor cell expression, and high affinity binding to Flk-1/KDR. J. Biol. Chem..

[177] DiSalvo J., Bayne M.L., Conn G., Kwok P.W., Trivedi P.G., Soderman D.D., Palisi T.M., Sullivan K.A., Thomas K.A. (1995). Purification and characterization of a naturally occurring vascular endothelial growth factor· placenta growth factor heterodimer. J. Biol. Chem..

[178] Ferrara N. (2004). Vascular endothelial growth factor: Basic science and clinical progress. Endocr. Rev..

[179] Park J.E., Chen H.H., Winer J., Houck K.A., Ferrara N. (1994). Placenta growth factor. Potentiation of vascular endothelial growth factor bioactivity, in vitro and in vivo, and high affinity binding to Flt-1 but not to Flk-1/KDR. J. Biol. Chem..

[180] Ferrara N. (2002). Role of vascular endothelial growth factor in physiologic and pathologic angiogenesis: Therapeutic implications. Semin. Oncol..

[181] Yoshiji H., Gomez D.E., Shibuya M., Thorgeirsson U.P. (1996). Expression of vascular endothelial growth factor, its receptor, and other angiogenic factors in human breast cancer. Cancer Res..

[182] Volm M., Koomägi R., Mattern J. (1997). Prognostic value of vascular endothelial growth factor and its receptor Flt-1 in squamous cell lung cancer. Int. J. Cancer.

[183] Ellis L., Takahashi Y., Fenoglio C., Cleary K., Bucana C., Evans D. (1998). Vessel counts and vascular endothelial growth factor expression in pancreatic adenocarcinoma. Eur. J. Cancer.

[184] Tomisawa M., Tokunaga T., Oshika Y., Tsuchida T., Fukushima Y., Sato H., Kijima H., Yamazaki H., Ueyama Y., Tamaoki N. (1999). Expression pattern of vascular endothelial growth factor isoform is closely correlated with tumour stage and vascularisation in renal cell carcinoma. Eur. J. Cancer.

[185] Sowter H., Corps A., Evans A., Clark D., Charnock-Jones D., Smith S. (1997). Expression and localization of the vascular endothelial growth factor family in ovarian epithelial tumors. Lab. Investig. A J. Tech. Methods Pathol..

[186] Ferrer F.A., Miller L.J., Andrawis R.I., Kurtzman S.H., Albertsen P.C., Laudone V.P., Kreutzer D.L. (1997). Vascular Endothelial Growth Factor (VEGF) Expression in Human Prostate Cancer: In Situ and in Vitro Expression of VEGF by Human Prostate Cancer Cells. J. Urol..

[187] Warren R.S., Yuan H., Matli M.R., Gillett N.A., Ferrara N. (1995). Regulation by vascular endothelial growth factor of human colon cancer tumorigenesis in a mouse model of experimental liver metastasis. J. Clin. Investig..

[188] Dias S., Hattori K., Heissig B., Zhu Z., Wu Y., Witte L., Hicklin D.J., Tateno M., Bohlen P., Moore M.A. (2001). Inhibition of both paracrine and autocrine VEGF/VEGFR-2 signaling pathways is essential to induce long-term remission of xenotransplanted human leukemias. Proc. Natl. Acad. Sci. USA.

[189] Mazurakova A., Samec M., Koklesova L., Biringer K., Kudela E., Al-Ishaq R.K., Pec M., Giordano F.A., Büsselberg D., Kubatka P. (2022). Anti-prostate cancer protection and therapy in the framework of predictive, preventive and personalised medicine-comprehensive effects of phytochemicals in primary, secondary and tertiary care. EPMA J..

[190] Bostwick D.G., Cooner W.H., Denis L., Jones G.W., Scardino P.T., Murphy G.P. (1992). The association of benign prostatic hyperplasia and cancer of the prostate. Cancer.

[191] Chokkalingam A.P., Nyrén O., Johansson J.E., Gridley G., McLaughlin J.K., Adami H.O., Hsing A.W. (2003). Prostate carcinoma risk subsequent to diagnosis of benign prostatic hyperplasia. Cancer.

[192] Schantz M.M., Bedner M., Long S.E., Molloy J.L., Murphy K.E., Porter B.J., Putzbach K., Rimmer C.A., Sander L.C., Sharpless K.E. (2008). Development of saw palmetto (Serenoa repens) fruit and extract standard reference materials. Anal. Bioanal. Chem..

[193] Gerber G.S., Fitzpatrick J.M. (2004). The role of a lipido-sterolic extract of Serenoa repens in the management of lower urinary tract symptoms associated with benign prostatic hyperplasia. BJU Int..

[194] Bonnar-Pizzorno R.M., Littman A.J., Kestin M., White E. (2006). Saw palmetto supplement use and prostate cancer risk. Nutr. Cancer.

[195] Bent S., Ko R. (2004). Commonly used herbal medicines in the United States: A review. Am. J. Med..

[196] Barnes P.M., Powell-Griner E., McFann K., Nahin R.L. (2004). Complementary and alternative medicine use among adults: United States, 2002. Seminars in Integrative Medicine.

[197] Bent S., Kane C., Shinohara K., Neuhaus J., Hudes E.S., Goldberg H., Avins A.L. (2006). Saw Palmetto for Benign Prostatic Hyperplasia. N. Engl. J. Med..

[198] Lowe F.C., Ku J.C. (1996). Phytotherapy in treatment of benign prostatic hyperplasia: A critical review. Urology.

[199] Goldmann W.H., Sharma A.L., Currier S.J., Johnston P.D., Rana A., Sharma C.P. (2001). Saw palmetto berry extract inhibits cell growth and Cox-2 expression in prostatic cancer cells. Cell Biol. Int..

[200] Iguchi K., Okumura N., Usui S., Sajiki H., Hirota K., Hirano K. (2001). Myristoleic acid, a cytotoxic component in the extract from Serenoa repens, induces apoptosis and necrosis in human prostatic LNCaP cells. Prostate.

[201] Hill B., Kyprianou N. (2004). Effect of permixon on human prostate cell growth: Lack of apoptotic action. Prostate.

[202] Mann J. (2002). Natural products in cancer chemotherapy: Past, present and future. Nat Rev Cancer.

[203] Katiyar C., Gupta A., Kanjilal S., Katiyar S. (2012). Drug discovery from plant sources: An integrated approach. Ayu.

[204] Monks A., Scudiero D., Skehan P., Shoemaker R., Paull K., Vistica D., Hose C., Langley J., Cronise P., Vaigro-Wolff A. (1991). Feasibility of a high-flux anticancer drug screen using a diverse panel of cultured human tumor cell lines. J. Natl. Cancer Inst..

[205] Ganesan A. (2008). The impact of natural products upon modern drug discovery. Curr. Opin. Chem. Biol..

[206] Bohn T., McDougall G.J., Alegría A., Alminger M., Arrigoni E., Aura A.-M., Brito C., Cilla A., El S.N., Karakaya S. (2015). Mind the gap—Deficits in our knowledge of aspects impacting the bioavailability of phytochemicals and their metabolites—A position paper focusing on carotenoids and polyphenols. Mol. Nutr. Food Res..

[207] Cordes E.H., Cordes E.H. (2020). 103C7The discovery of taxol: Wall, Horwitz, Holton. Hallelujah Moments: Tales of Drug Discovery.

[208] Barani M., Sangiovanni E., Angarano M., Rajizadeh M.A., Mehrabani M., Piazza S., Gangadharappa H.V., Pardakhty A., Mehrbani M., Dell’Agli M. (2021). Phytosomes as Innovative Delivery Systems for Phytochemicals: A Comprehensive Review of Literature. Int. J. Nanomed..

[209] Suvarna V., Gujar P., Murahari M. (2017). Complexation of phytochemicals with cyclodextrin derivatives—An insight. Biomed. Pharmacother..

[210] Li J., Wang Y., Yang C., Wang P., Oelschlager D.K., Zheng Y., Tian D.A., Grizzle W.E., Buchsbaum D.J., Wan M. (2009). Polyethylene glycosylated curcumin conjugate inhibits pancreatic cancer cell growth through inactivation of Jab1. Mol. Pharmacol..

[211] Rijo P., Matias D., Fernandes A., Simões M., Nicolai M., Reis C. (2014). Antimicrobial Plant Extracts Encapsulated into Polymeric Beads for Potential Application on the Skin. Polymers.

[212] Date A.A., Hanes J., Ensign L.M. (2016). Nanoparticles for oral delivery: Design, evaluation and state-of-the-art. J. Control Release.

[213] Xie J., Yang Z., Zhou C., Zhu J., Lee R.J., Teng L. (2016). Nanotechnology for the delivery of phytochemicals in cancer therapy. Biotechnol. Adv..

[214] Huang Q., Yu H., Ru Q. (2010). Bioavailability and Delivery of Nutraceuticals Using Nanotechnology. J. Food Sci..

[215] Chan J., Elkin E., Silva S., Broering J., Latini D., Carroll P. (2005). Total and specific complementary and alternative medicine use in a large cohort of men with prostate cancer. Urology.

[216] Lee M.M., Chang J.S., Jacobs B., Wrensch M.R. (2002). Complementary and alternative medicine use among men with prostate cancer in 4 ethnic populations. Am. J. Public Health.

[217] Trottier G., Bostrom P.J., Lawrentschuk N., Fleshner N.E. (2010). Nutraceuticals and prostate cancer prevention: A current review. Nat Rev Urol.

[218] Curtis Nickel J., Shoskes D., Roehrborn C.G., Moyad M. (2008). Nutraceuticals in Prostate Disease: The Urologist’s Role. Rev. Urol..

[219] Coppens P., Da Silva M.F., Pettman S. (2006). European regulations on nutraceuticals, dietary supplements and functional foods: A framework based on safety. Toxicology.

[220] Arora M., Sharma S., Baldi A. (2013). Comparative insight of regulatory guidelines for probiotics in USA, India and Malaysia: A critical review. Int. J. Biotechnol. Wellness Ind..

[221] Nelson V.K., Pullaiah C.P., Saleem Ts M., Roychoudhury S., Chinnappan S., Vishnusai B., Ram Mani R., Birudala G., Bottu K.S. (2022). Natural Products as the Modulators of Oxidative Stress: An Herbal Approach in the Management of Prostate Cancer. Adv. Exp. Med. Biol..

[222] Singh K., Bhori M., Kasu Y.A., Bhat G., Marar T. (2018). Antioxidants as precision weapons in war against cancer chemotherapy induced toxicity—Exploring the armoury of obscurity. Saudi Pharm. J..

[223] Anon, Industry M.o.A.a.A. (2016). National Agrofood Policy 2011–2020. https://ap.fftc.org.tw/article/1368.

[224] Akarasereenont P., Datiles M.J.R., Lumlerdkij N., Yaakob H., Prieto J.M., Heinrich M. (2015). A South-East Asian Perspective on Ethnopharmacology. Ethnopharmacology.

[225] Idris M.K.H., Hasham R., Ismail H.F. (2022). Bioassay-Guided extraction of andrographis paniculata for intervention of in-vitro prostate cancer progression in metabolic syndrome environment. Daru.

[226] Vetvicka V., Vannucci L. (2021). Biological properties of andrographolide, an active ingredient of Andrographis Paniculata: A narrative review. Ann. Transl. Med..

[227] Mir H., Kapur N., Singh R., Sonpavde G., Lillard J.W., Singh S. (2016). Andrographolide inhibits prostate cancer by targeting cell cycle regulators, CXCR3 and CXCR7 chemokine receptors. Cell Cycle.

[228] Zhao F., He E.Q., Wang L., Liu K. (2008). Anti-tumor activities of andrographolide, a diterpene from Andrographis paniculata, by inducing apoptosis and inhibiting VEGF level. J. Asian Nat. Prod. Res..

[229] Chun J.Y., Tummala R., Nadiminty N., Lou W., Liu C., Yang J., Evans C.P., Zhou Q., Gao A.C. (2010). Andrographolide, an herbal medicine, inhibits interleukin-6 expression and suppresses prostate cancer cell growth. Genes Cancer.

[230] Forestier-Román I.S., López-Rivas A., Sánchez-Vázquez M.M., Rohena-Rivera K., Nieves-Burgos G., Ortiz-Zuazaga H., Torres-Ramos C.A., Martínez-Ferrer M. (2019). Andrographolide induces DNA damage in prostate cancer cells. Oncotarget.

[231] Choi Y.-J., Fan M., Wedamulla N.E., Tang Y., Bae S.M., Hwang J.-Y., Kim E.-K. (2022). Inhibitory effects of *Centella asiatica* (L.) Urban on enlarged prostate through androgen receptor and PI3K/Akt signaling pathways. Food Funct..

[232] Alafnan A., Hussain T., Rizvi S.M.D., Moin A., Alamri A. (2021). Prostate Apoptotic Induction and NFκB Suppression by Dammarolic Acid: Mechanistic Insight into Onco-Therapeutic Action of an Aglycone Asiaticoside. Curr. Issues Mol. Biol..

[233] Hsu Y.L., Kuo P.L., Lin L.T., Lin C.C. (2005). Asiatic acid, a triterpene, induces apoptosis and cell cycle arrest through activation of extracellular signal-regulated kinase and p38 mitogen-activated protein kinase pathways in human breast cancer cells. J. Pharmacol. Exp. Ther..

[234] Adtani P.N., Narasimhan M., Punnoose A.M., Kambalachenu H.R. (2017). Antifibrotic effect of Centella asiatica Linn and asiatic acid on arecoline-induced fibrosis in human buccal fibroblasts. J. Investig. Clin. Dent..

[235] Hao Y., Huang J., Ma Y., Chen W., Fan Q., Sun X., Shao M., Cai H. (2018). Asiatic acid inhibits proliferation, migration and induces apoptosis by regulating Pdcd4 via the PI3K/Akt/mTOR/p70S6K signaling pathway in human colon carcinoma cells. Oncol. Lett..

[236] Zulkipli I.N., Rajabalaya R., Idris A., Sulaiman N.A., David S.R. (2017). Clinacanthus nutans: A review on ethnomedicinal uses, chemical constituents and pharmacological properties. Pharm. Biol..

[237] Teoh P.L., Cheng A.Y., Liau M., Lem F.F., Kaling G.P., Chua F.N., Cheong B.E. (2017). Chemical composition and cytotoxic properties of Clinacanthus nutans root extracts. Pharm. Biol..

[238] Ng P.Y., Chye S.M., Ng Ch H., Koh R.Y., Tiong Y.L., Pui L.P., Tan Y.H., Lim C.S., Ng Kh Y. (2017). Clinacanthus Nutans Hexane Extracts Induce Apoptosis Through a Caspase-Dependent Pathway in Human Cancer Cell Lines. Asian Pac. J. Cancer Prev..

[239] Md Toha Z., Haron N.H., Kamal N.N.S.N.M., Khairuddean M., Arsad H. (2022). Exploring Clinacanthus nutans leaf different solvent extracts on antiproliferative effects induced metastasis through apoptosis and cell cycle against MCF-7 human breast cancer cell lines. Future J. Pharm. Sci..

[240] Fazil F.N., Azzimi N.S., Yahaya B.H., Kamalaldin N.A., Zubairi S.I. (2016). Kinetics Extraction Modelling and Antiproliferative Activity of Clinacanthus nutans Water Extract. Sci. World J..

[241] George A., Henkel R. (2014). Phytoandrogenic properties of Eurycoma longifolia as natural alternative to testosterone replacement therapy. Andrologia.

[242] Tong K.L., Chan K.L., AbuBakar S., Low B.S., Ma H.Q., Wong P.F. (2015). The in vitro and in vivo anti-cancer activities of a standardized quassinoids composition from Eurycoma longifolia on LNCaP human prostate cancer cells. PloS ONE.

[243] Berg C. (2003). Flora Malesiana precursor for the treatment of Moraceae 3: Ficus subgenus Ficus. Blumea-Biodivers. Evol. Biogeogr. Plants.

[244] Burkill I.H., Haniff M. (1930). Malay Village Medicine: Prescriptions Collected by.

[245] Abdullah N., Karsani S., Aminudin N. (2008). Effects of Ficus Deltoidea Extract on the Serum Protein Profile of Simultaneously Hypertensive Rats (SHR). J. Proteom. Bioinform. (JPB).

[246] Hadijah H., Normah A., Ahmad Tarmizi S., Aida M. Cholesterol lowering effect of mas cotek tea in hypercholesterolemic rats. Proceedings of the 2nd International Conference of East-West Perspective of Functional Food Science.

[247] Bunawan H., Amin N.M., Bunawan S.N., Baharum S.N., Mohd Noor N. (2014). Ficus deltoidea Jack: A Review on Its Phytochemical and Pharmacological Importance. Evid.-Based Complement. Altern. Med..

[248] Zakaria Z.A., Hussain M.K., Mohamad A.S., Abdullah F.C., Sulaiman M.R. (2012). Anti-Inflammatory Activity of the Aqueous Extract of Ficus Deltoidea. Biol. Res. Nurs..

[249] Abdullah Z., Hussain K., Ismail Z., Ali R.M. (2009). Anti-inflammatory activity of standardised extracts of leaves of three varieties of Ficus deltoidea. Asian J. Pharm. Clin. Res..

[250] Uyub A.M., Nwachukwu I.N., Azlan A.A., Fariza S.S. (2010). In-vitro antibacterial activity and cytotoxicity of selected medicinal plant extracts from Penang Island Malaysia on metronidazole-resistant-Helicobacter pylori and some pathogenic bacteria. Ethnobot. Res. Appl..

[251] Akhir N.A.M., Chua L.S., Majid F.A.A., Sarmidi M.R. (2011). Cytotoxicity of aqueous and ethanolic extracts of Ficus deltoidea on human ovarian carcinoma cell line. Br. J. Med. Med. Res..

[252] Zhou Y., Liu Y.E., Cao J., Zeng G., Shen C., Li Y., Zhou M., Chen Y., Pu W., Potters L. (2009). Vitexins, nature-derived lignan compounds, induce apoptosis and suppress tumor growth. Clin. Cancer Res..

[253] Lin J.P., Yang J.S., Lin J.J., Lai K.C., Lu H.F., Ma C.Y., Sai-Chuen Wu R., Wu K.C., Chueh F.S., Gibson Wood W. (2012). Rutin inhibits human leukemia tumor growth in a murine xenograft model in vivo. Environ. Toxicol..

[254] Zheng S.-Y., Li Y., Jiang D., Zhao J., Ge J.-F. (2012). Anticancer effect and apoptosis induction by quercetin in the human lung cancer cell line A-549. Mol. Med. Rep..

[255] Guo Q., Tian X., Yang A., Zhou Y., Wu D., Wang Z. (2014). Orientin in Trollius chinensis Bunge inhibits proliferation of HeLa human cervical carcinoma cells by induction of apoptosis. Mon. Für Chem.-Chem. Mon..

[256] Chiang Y.-M., Kuo Y.-H. (2002). Novel Triterpenoids from the Aerial Roots of Ficus m icrocarpa. J. Org. Chem..

[257] Chiang Y.-M., Chang J.-Y., Kuo C.-C., Chang C.-Y., Kuo Y.-H. (2005). Cytotoxic triterpenes from the aerial roots of *Ficus microcarpa*. Phytochemistry.

[258] Hanafi M.M.M., Afzan A., Yaakob H., Aziz R., Sarmidi M.R., Wolfender J.L., Prieto J.M. (2017). In Vitro Pro-apoptotic and Anti-migratory Effects of Ficus deltoidea L. Plant Extracts on the Human Prostate Cancer Cell Lines PC3. Front. Pharmacol..

[259] Worawattananutai P., Itharat A., Ruangnoo S. (2014). In vitro antioxidant, anti-inflammatory, cytotoxic activities against prostate cancer of extracts from Hibiscus sabdariffa leaves. J. Med. Assoc. Thail..

[260] Chiu C.T., Chen J.H., Chou F.P., Lin H.H. (2015). Hibiscus sabdariffa Leaf Extract Inhibits Human Prostate Cancer Cell Invasion via Down-Regulation of Akt/NF-kB/MMP-9 Pathway. Nutrients.

[261] Song B., Shen X., Tong C., Zhang S., Chen Q., Li Y., Li S. (2023). Gossypin: A flavonoid with diverse pharmacological effects. Chem. Biol. Drug Des..

[262] Chen D.L., Ma G.X., Yang E.L., Yang Y., Wang C.H., Sun Z.C., Liang H.Q., Xu X.D., Wei J.H. (2022). Cadinane-type sesquiterpenoid dimeric diastereomers hibisceusones A-C from infected stems of Hibiscus tiliaceus with cytotoxic activity against triple-negative breast cancer cells. Bioorganic Chem..

[263] Rasul H.O., Aziz B.K., Ghafour D.D., Kivrak A. (2022). Screening the possible anti-cancer constituents of Hibiscus rosa-sinensis flower to address mammalian target of rapamycin: An in silico molecular docking, HYDE scoring, dynamic studies, and pharmacokinetic prediction. Mol. Divers..

[264] Laskar Y.B., Mazumder P.B., Talukdar A.D. (2023). Hibiscus sabdariffa anthocyanins are potential modulators of estrogen receptor alpha activity with favourable toxicology: A computational analysis using molecular docking, ADME/Tox prediction, 2D/3D QSAR and molecular dynamics simulation. J. Biomol. Struct. Dyn..

[265] Jamal J.A., Houghton P., Milligan S., Jantan I. (2003). The Oestrogenis and cytotoxic effects of the extracts of Labisia pumila var. alata and Labisia pumila var. pumila in vitro. Malays. J. Health Sci..

[266] Ibrahim M.H., Jaafar H.Z. (2011). Photosynthetic capacity, photochemical efficiency and chlorophyll content of three varieties of Labisia pumila Benth. exposed to open field and greenhouse growing conditions. Acta Physiol. Plant..

[267] Al-Mekhlafi N.A., Shaari K., Abas F., Kneer R., Jeyaraj E.J., Stanslas J., Yamamoto N., Honda T., Lajis N.H. (2012). Alkenylresorcinols and cytotoxic activity of the constituents isolated from *Labisia pumila*. Phytochemistry.

[268] Chua L.S., Lee S.Y., Abdullah N., Sarmidi M.R. (2012). Review on *Labisia pumila* (Kacip Fatimah): Bioactive phytochemicals and skin collagen synthesis promoting herb. Fitoterapia.

[269] Chen H.M., Wu Y.C., Chia Y.C., Chang F.R., Hsu H.K., Hsieh Y.C., Chen C.C., Yuan S.S. (2009). Gallic acid, a major component of Toona sinensis leaf extracts, contains a ROS-mediated anti-cancer activity in human prostate cancer cells. Cancer Lett..

[270] Albrecht D.S., Clubbs E.A., Ferruzzi M., Bomser J.A. (2008). Epigallocatechin-3-gallate (EGCG) inhibits PC-3 prostate cancer cell proliferation via MEK-independent ERK1/2 activation. Chem.-Biol. Interact..

[271] Pravettoni A., Mornati O., Martini P., Marino M., Colciago A., Celotti F., Motta M., Negri-Cesi P. (2007). Estrogen receptor beta (ERbeta) and inhibition of prostate cancer cell proliferation: Studies on the possible mechanism of action in DU145 cells. Mol. Cell. Endocrinol..

[272] Adlercreutz H. (2002). Phyto-oestrogens and cancer. Lancet Oncol..

[273] Hussain M., Banerjee M., Sarkar F.H., Djuric Z., Pollak M.N., Doerge D., Fontana J., Chinni S., Davis J., Forman J. (2003). Soy isoflavones in the treatment of prostate cancer. Nutr. Cancer.

[274] Mohd Hanafi M.M., Yaakob H., Sarmidi M.R., Aziz R., Prieto J.M. (2016). Marantodes pumilum L. plant extracts induce apoptosis, cell cycle arrest and inhibit cell migration and invasion on prostate cancer cell lines. Planta Med..

[275] Hirasawa Y., Pagano I., Huang J., Sasaki Y., Murakami K., Rosser C.J., Furuya H. (2021). Case Study of Noni Extract in Men with Very Low-Risk or Low-Risk Prostate Cancer. Hawaii J. Health Soc. Welf..

[276] Akihisa T., Matsumoto K., Tokuda H., Yasukawa K., Seino K., Nakamoto K., Kuninaga H., Suzuki T., Kimura Y. (2007). Anti-inflammatory and potential cancer chemopreventive constituents of the fruits of Morinda citrifolia (Noni). J. Nat. Prod..

[277] Liu G., Bode A., Ma W.Y., Sang S., Ho C.T., Dong Z. (2001). Two novel glycosides from the fruits of Morinda citrifolia (noni) inhibit AP-1 transactivation and cell transformation in the mouse epidermal JB6 cell line. Cancer Res..

[278] Hiwasa T., Arase Y., Chen Z., Kita K., Umezawa K., Ito H., Suzuki N. (1999). Stimulation of ultraviolet-induced apoptosis of human fibroblast UVr-1 cells by tyrosine kinase inhibitors. FEBS Lett..

[279] Liu X.L., Zhang L., Fu X.L., Chen K., Qian B.C. (2001). Effect of scopoletin on PC3 cell proliferation and apoptosis. Acta Pharmacol. Sin..

[280] Carastro L.M., Vallebuona E.J., Cordova R., Gannon A.N., Kim S.J., Costello C.M., Declet-Bauzo R.A., Kumar N., Park J.Y. (2022). Polyphenon E Effects on Gene Expression in PC-3 Prostate Cancer Cells. Int. J. Mol. Sci..

[281] Patel K., Patel D.K. (2022). Therapeutic effectiveness of sinensetin against cancer and other human complications: A review of biological potential and pharmacological activities. Cardiovasc. Hematol. Disord. Drug Targets.

[282] Suhaimi S.H., Hasham R., Hafiz Idris M.K., Ismail H.F., Mohd Ariffin N.H., Abdul Majid F.A. (2019). Optimization of Ultrasound-Assisted Extraction Conditions Followed by Solid Phase Extraction Fractionation from Orthosiphon stamineus Benth (Lamiace) Leaves for Antiproliferative Effect on Prostate Cancer Cells. Molecules.

[283] Al-Suede F.S., Khadeer Ahamed M.B., Abdul Majid A.S., Baharetha H.M., Hassan L.E., Kadir M.O., Nassar Z.D., Abdul Majid A.M. (2014). Optimization of Cat’s Whiskers Tea (Orthosiphon stamineus) Using Supercritical Carbon Dioxide and Selective Chemotherapeutic Potential against Prostate Cancer Cells. Evid.-Based Complement. Altern. Med. Ecam.

[284] Manthey J.A., Guthrie N. (2002). Antiproliferative activities of citrus flavonoids against six human cancer cell lines. J. Agric. Food Chem..

[285] Tang Y.Q., Jaganath I., Manikam R., Sekaran S.D. (2013). Phyllanthus Suppresses Prostate Cancer Cell, PC-3, Proliferation and Induces Apoptosis through Multiple Signalling Pathways (MAPKs, PI3K/Akt, NFκB, and Hypoxia). Evid.-Based Complement. Altern. Med. Ecam.

[286] Tang Y.Q., Jaganath I.B., Sekaran S.D. (2010). Phyllanthus spp. induces selective growth inhibition of PC-3 and MeWo human cancer cells through modulation of cell cycle and induction of apoptosis. PloS ONE.

[287] Mohd Hanafi M. (2017). In Vitro Pro-Apoptotic and Anti-Migratory Effects of Marantodes Pumilum (Blume) Kuntze and Ficus Deltoidea L. Extracts on Prostate Cancer Cell Lines. Ph.D. Thesis.

